# Supplementary material for: Bi-allelic JAM2 Variants Lead to Early-Onset Recessive Primary Familial Brain Calcification
Source: Am J Hum Genet. 2020 Mar 5;106(3):412–21. doi: 10.1016/j.ajhg.2020.02.007 (PMC7058839; doi:10.1016/j.ajhg.2020.02.007)
Supplement: Document S1. Figures S1–S7 [file mmc1.pdf]

The American Journal of Human Genetics, Volume *106*

Supplemental Data

Bi-allelic *JAM2* Variants Lead to Early-Onset Recessive Primary Familial Brain Calcification

Lucia V. Schottlaender, Rosella Abeti, Zane Jaunmuktane, Carol Macmillan, Viorica Chelban, Benjamin O'Callaghan, John McKinley, Reza Maroofian, Stephanie Efthymiou, Alkyoni Athanasiou-Fragkouli, Raeburn Forbes, Marc P.M. Soutar, John H. Livingston, Bernardett Kalmar, Orlando Swayne, Gary Hotton, SYNAPS Study Group, Alan Pittman, João Ricardo Mendes de Oliveira, Maria de Grandis, Angela Richard-Loendt, Francesca Launchbury, Juri Althonayan, Gavin McDonnell, Aisling Carr, Suliman Khan, Christian Beetz, Atil Bisgin, Sevcan Tug Bozdogan, Amber Begtrup, Erin Torti, Linda Greensmith, Paola Giunti, Patrick J. Morrison, Sebastian Brandner, Michel Aurrand-Lions, and Henry Houlden

Supplemental data

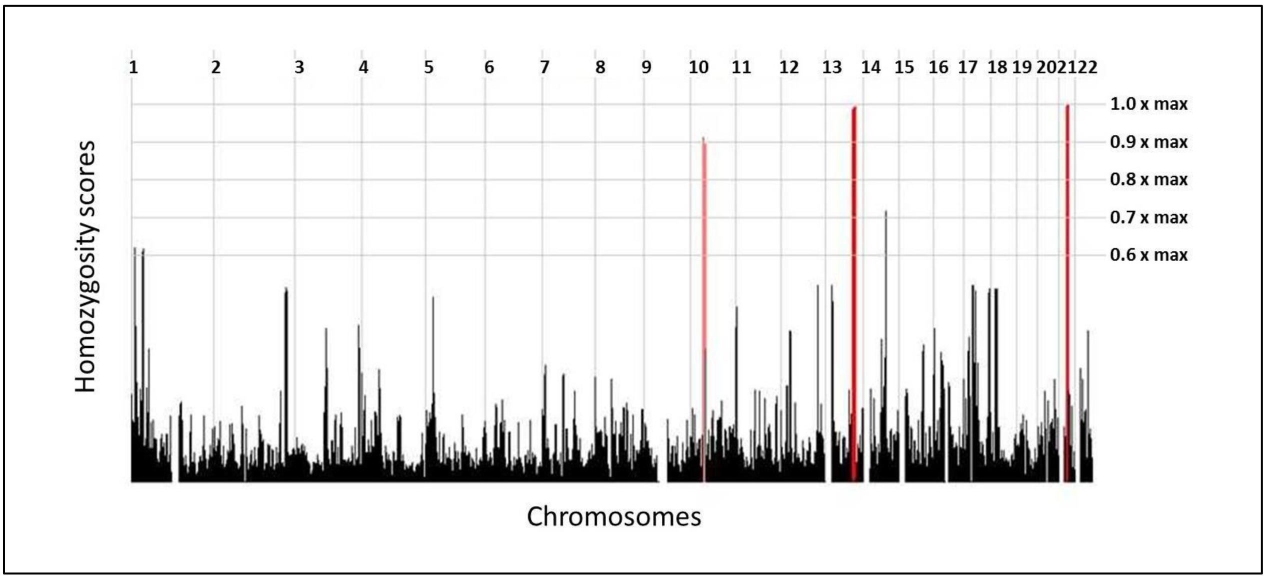

Figure S1: Genome wide illustration of homozygous regions. Three significant regions of homozygosity were detected: Chr 10: 37414883-43132376; Chr 13: 88327643-93518692; and Chr 21: 22370881-28338710 and they are highlighted in red.

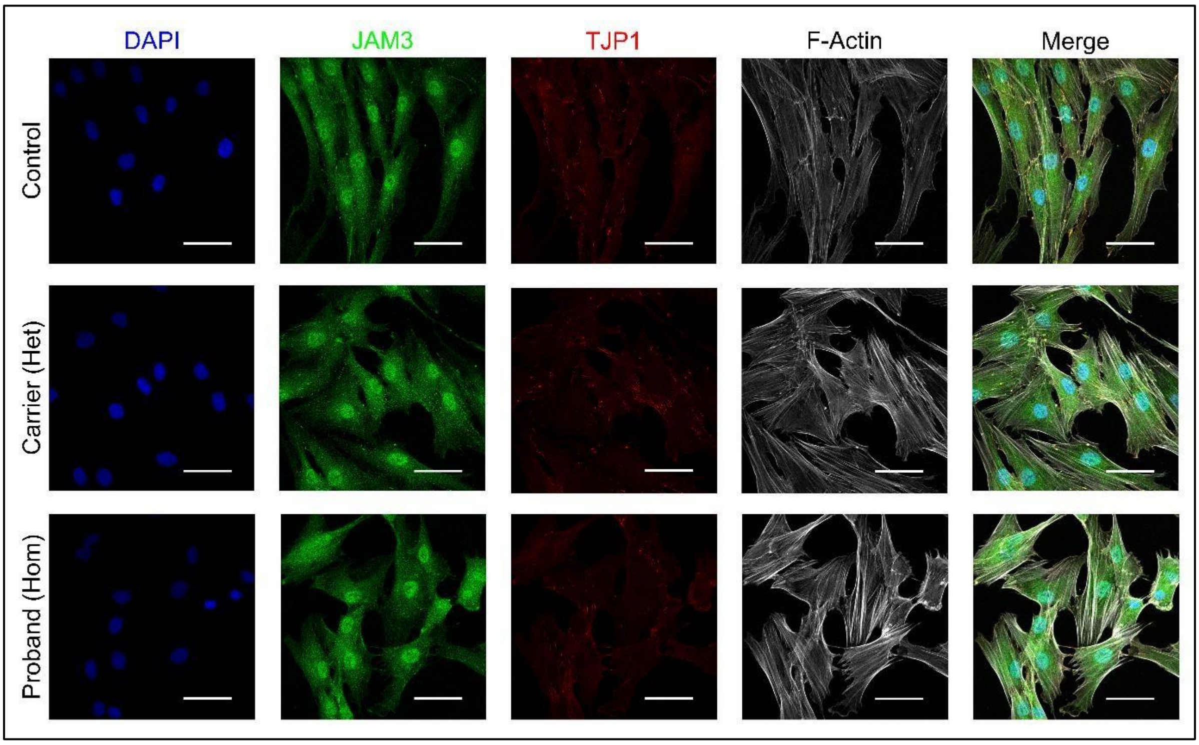

Figure S2: Localisation of JAM3 and TJP1 tight junction proteins in primary dermal fibroblasts from the proband of Family 1 (Hom) and his unaffected mother (Het). Scale bars = 50 μm.

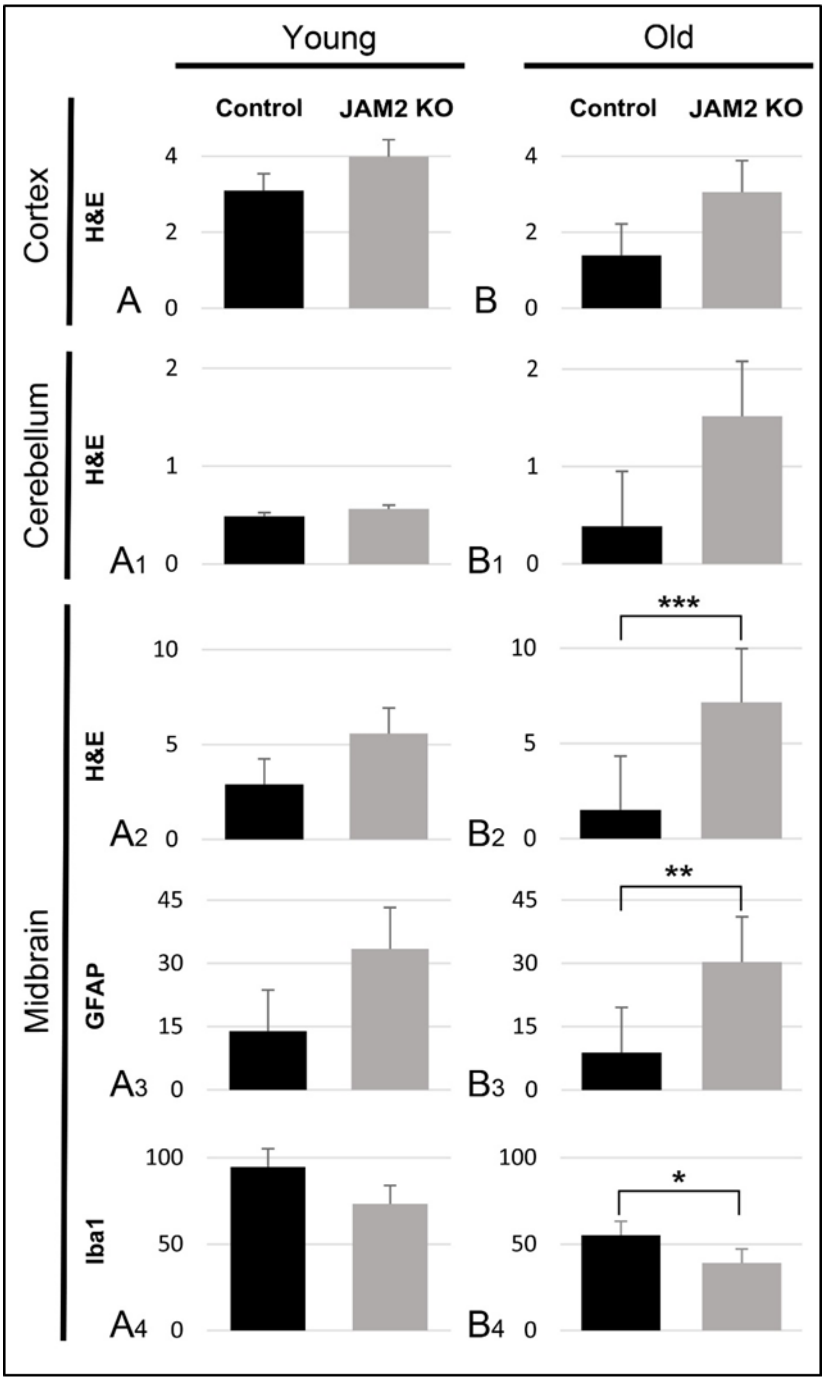

Figure S3: Quantitative data of neuropil vacuolation, astrogliosis and microglial activation in young and old *jam*2 KO and wild-type (Control) mice. The automated quantification of the neuropil vacuolation on H&E stained sections (A, A1, A2, B, B1, B2), GFAP immunoreactive gliosis (A3, B3) and Iba1 positive microglial activation (A4, B4) in young (A-A4) and aged (B-B4) wild-type (black columns) and *jam2* KO (grey columns) mice. Automated quantification of the percentage of vacuolation, gliosis and microglial activation in

selected areas (cortex, midbrain and cerebellum) was performed on digitalised slides, using open source software QuPath.

In young *jam2* KO mice (n=2) when compared with age-matched wild-type mice (n=2), there was a trend towards greater neuropil vacuolation in the cortex (A), cerebellum (A1) and midbrain (A2). Similarly, GFAP positive astrogliosis (A3), but not Iba1 positive microglial activation (A4), was more prominent in young *jam2* KO mice, than wild-type mice. In the aged *jam2* KO mice (n=4), there was a trend towards greater neuropil vacuolation in the cortex (B) and cerebellum (B1), when compared with age-matched wild-type mice (n=4). There was a significant increase in the degree of neuropil vacuolation (*** $p<0.00007$) and astrogliosis (**$p<0.0138$) in the midbrain (B3) of old *jam2* KO mice when compared with age-matched wild-type mice, whereas Iba1 positive microglial activation (B4) in old *jam2* KO mice was less pronounced than in age-matched wild-type mice (*$p<0.035$).

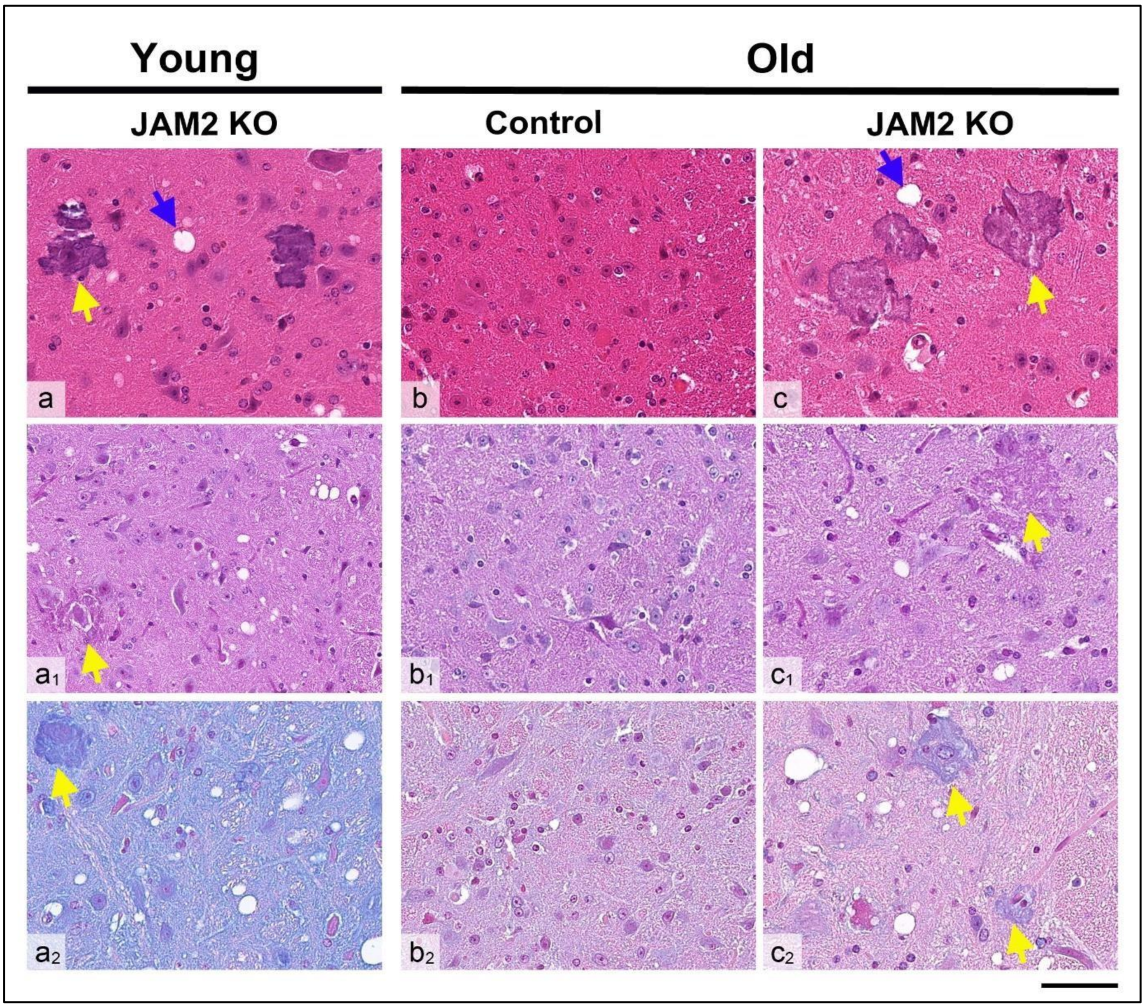

Figure S4: Spinal cord pathology in control mice and *jam2* KO mice of young (6 months) and old (18 months) age. In the grey matter of young *jam2* KO mice (a, a1 and a2) (a1) there is frequent mineralisation (yellow arrow) and occasional vacuolation in the neuropil (blue arrow). The mineralised deposits show no reactivity with PAS tinctorial stain (a1, yellow arrow) and very weak labelling with Alcian blue (a2, yellow arrow). In the spinal cord grey matter of old control wild-type mice (b, b1 and b2) there is no mineralisation and no apparent neuropil vacuolation. In the spinal cord of an old *jam2* KO mouse (c, c1 and c2), however, there is frequent mineralisation (c, yellow arrow) and vacuolation in the neuropil (blue arrow) involving both anterior and posterior horns. The mineralised deposits also in old *jam2* KO mice are not positive for PAS (c1) and weakly label for Alcian blue (c2, one yellow arrow

accentuates a mineralised neurone and the other shows perivascular mineralisation). Scale bar: 100µm a-c, a1-c1, a2-c2.

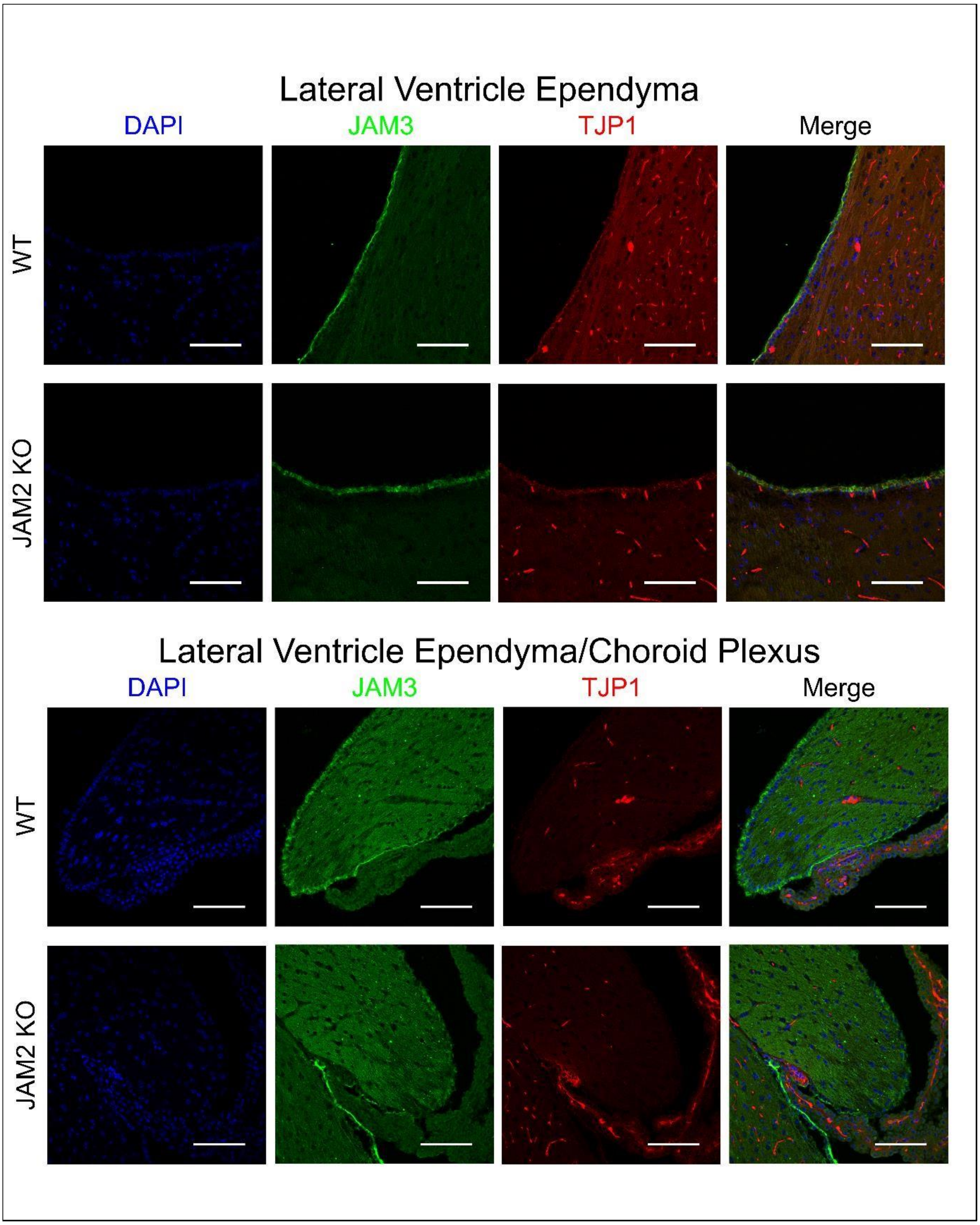

Figure S5: Correct localisation of JAM3 and TJP1 tight-junction proteins in the lateral ventricle ependyma of *jam2* KO and wild-type (WT) mice. Scale bars = 100 µm.

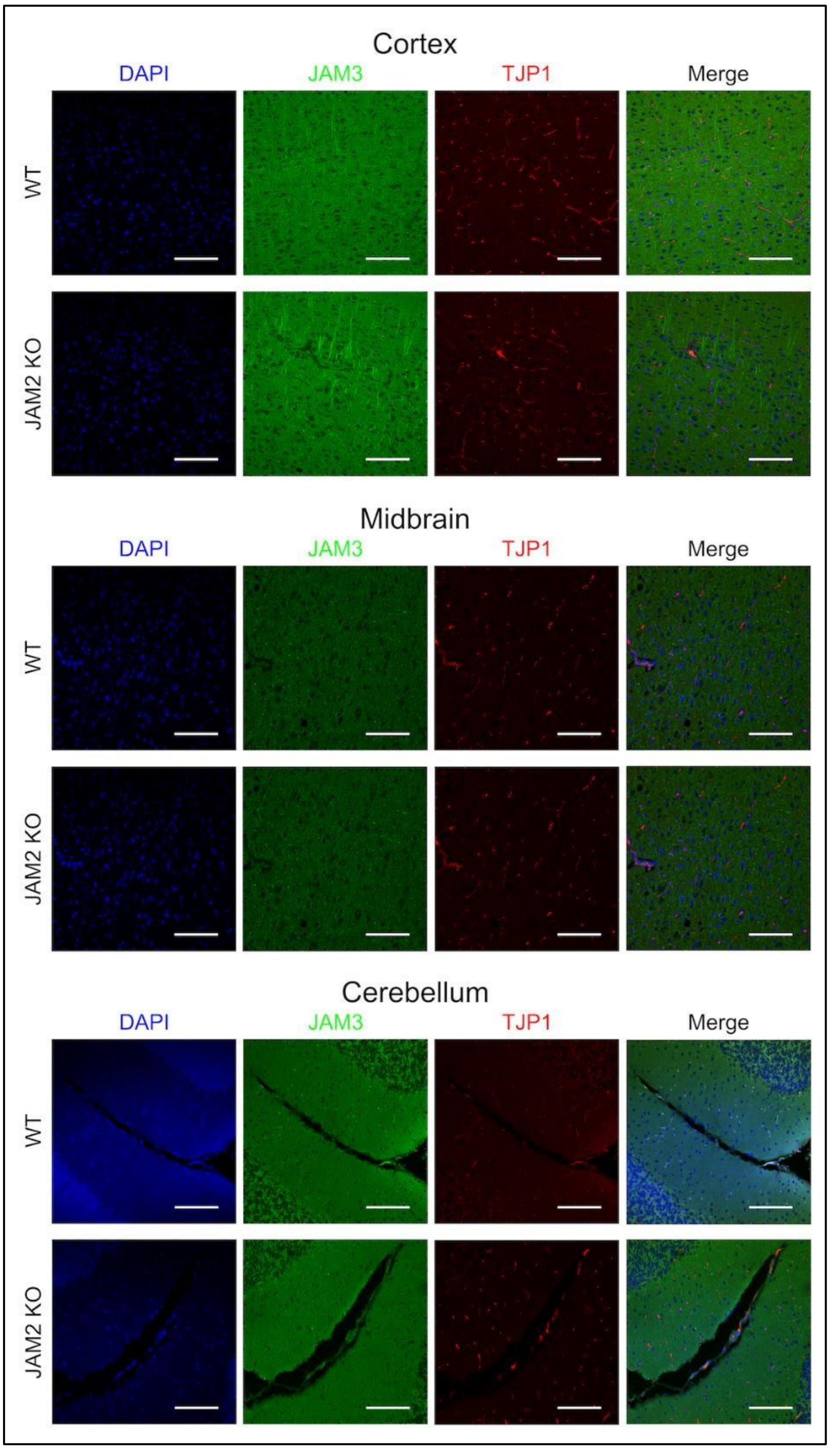

Figure S6: Unaltered staining pattern for JAM3 and TJP1 tight-junction proteins in different brain regions of *jam2* KO and wild-type (WT) mice. Scale bars = 100 μm.

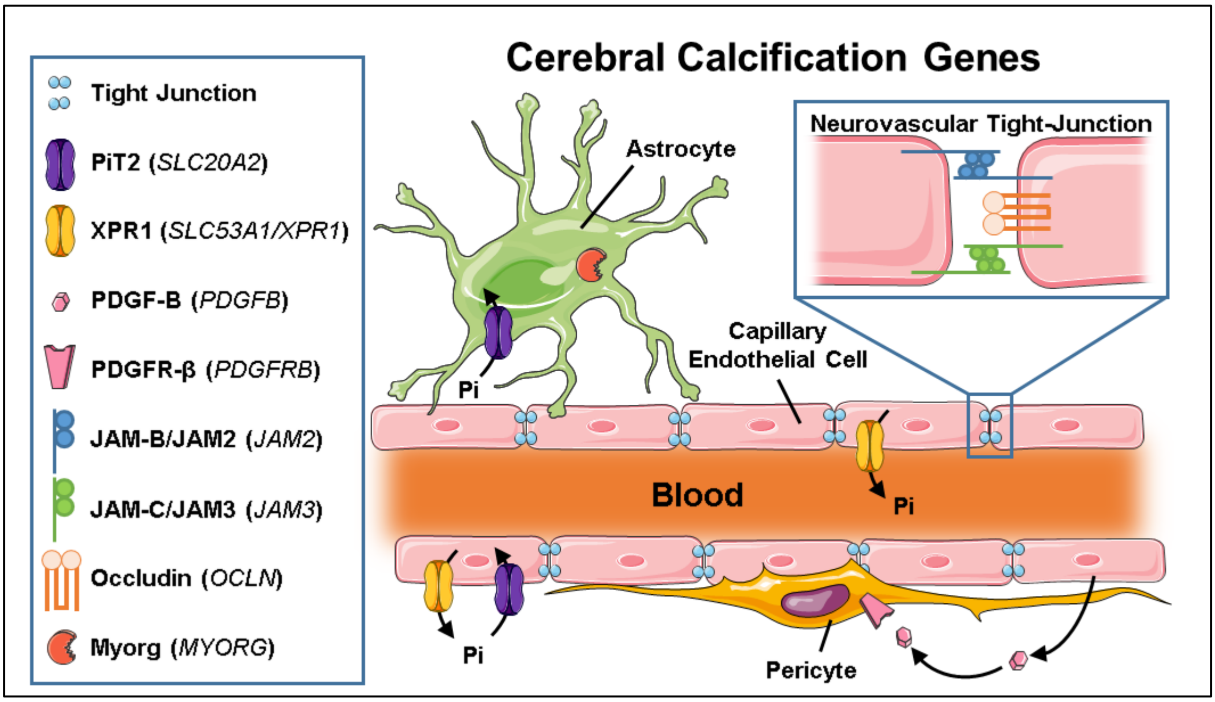

Figure S7: Schematic highlighting the contributions of proteins associated with brain calcification disorders in cell types of the central neurovascular unit (NVU). Autosomal dominant mutations in genes encoding the inorganic phosphate transporter 2; (PiT2 encoded by *SLC20A2*) and inorganic phosphate exporter; (XPR1 encoded by *SLC53A1/XPR1*) are causative of PFBC, highlighting dysregulation of phosphate homeostasis as an important cause of calcium deposition. Platelet-derived growth factor subunit B (PDGF-B encoded by *PDGFB*) signalling through the platelet-derived growth factor receptor-β (PDGFR-β encoded by *PDGFRB*) is important for pericyte survival and blood brain barrier (BBB) maintenance. Autosomal dominant mutations in *PDGFB* and *PDGFRB* cause PFBC suggesting loss of BBB integrity as an additional pathomechanism. In line with this, recessive mutations in *JAM3* and *OCLN* encoding tight junction proteins have been associated with complex neurological disorders that include brain calcification. The *JAM2* mutations identified in this study further support dysregulation of BBB integrity as a PFBC pathomechanism. This figure was prepared using Servier Medical Art, licensed under a Creative Common Attribution 3.0 Generic License (http://smart.servier.com/).

Supplemental methods

This study was approved by UCLH Queen Square ethics committee and local institutional review board (UCLH: 04/N034) and informed consent was obtained from all subjects or their legal guardians when applicable.

Genome-wide genotyping and homozygosity mapping

Genomic DNA was extracted from peripheral blood samples. In family 2 genome-wide genotyping was performed according to the manufacturers' instructions on Illumina HumanCytoSNP-12v2-1_H array, which contains probes for over 200,000 markers distributed across the genome. Default parameters on genome studio software 2010.3 (Illumina) were used for clustering and normalization of raw data, and to obtain genotype calls. Regions of homozygosity were then analysed with homozygosity mapper software[1].

Exome sequencing (ES)

ES was performed in all families. In familied 1 and 2 exome sequencing was done with Illumina® Nextera focused exome capture on a HiSeq 2000. Paired-end sequence reads were aligned with Novoalign against the reference human genome (UCSC hg19). Duplicate read removal, format conversion, and indexing were performed with Picard. The Genome Analysis Toolkit (GATK) was used to recalibrate base quality scores, perform local realignments around possible indels, and to call and filter the variants. Annotated variant files were generated using ANNOVAR[2]. ES in family 3 was carried by GeneDx. Using genomic DNA from the proband and parents, the exonic regions and flanking splice junctions of the genome were captured using the Clinical Research Exome kit (Agilent Technologies, Santa Clara, CA). Massively parallel (NextGen) sequencing was done on an Illumina system with 100bp or greater paired-end reads. Reads were aligned to human genome build GRCh37/UCSC hg19, and analyzed for sequence

variants using a custom-developed analysis tool. Additional sequencing technology and variant interpretation protocol has been previously described[3]. The general assertion criteria for variant classification are publicly available on the GeneDx ClinVar submission page (http://www.ncbi.nlm.nih.gov/clinvar/submitters/26957/). ES in the proband of family 4 was performed using the capture system from Twist Human Core Exome Plus kit (Twist Bioscience®) and sequenced on a Illumina HiSeqX that produced 150-bp paired-end reads. Sequencing data were aligned using BWA software and alignments were converted to binary bam file format with GATK. For variant calling we utilized the GATK HaplotypeCaller. Additional sequencing and bioinformatics information on this case was previously described[4].

Cell culture

Fibroblasts from one proband and his mother from Family 1 and unrelated controls were cultured from a skin biopsy in Dulbecco's modified Eagle medium containing 4.5 g/L glucose and supplemented with 10 % heat-inactivated fetal calf serum and maintained in a humidified chamber at 37 °C with 5 % CO2.

RT-PCR

RNA was extracted from human fibroblasts and purified using the Quick-RNA Miniprep Kit (Zymo Research). A complementary DNA (cDNA) library was immediately created from 500 ng of the RNA obtained, by reverse transcription reaction with Superscript II reverse transcriptase (Thermofisher). Reverse transcription PCR (RT-PCR) was then performed using equal volumes of cDNA using FastStart Taq DNA polymerase master mix and primer pairs specific for GAPDH (loading control) and *JAM2* (Supplementary Table 1). Equal volumes of the PCR mixes were then separated by DNA gel electrophoresis through 1.2% w/v agarose TAE. Dye-terminator sequencing reactions were performed using the BigDye® Terminator

v3.1 Cycle Sequencing Kit (Applied Biosystems) and analysed on a 3730xl DNA Analyser (Applied Biosystems).

The primers used for RT-PCR were:

**Target**	**Forward Primer (5'-3')**	**Reverse Primer (5'-3')**	**Product (bp)**
GAPDH	TGGTGCTGAGTATGTCGTGGAGT	AGTCTTCTGAGTGGCAGTGATGG	292
JAM2	GTGACAAGAAGTGATGCGGG	GCATAGCATACACCAAGGCC	488

SDS-PAGE and Western blotting

Cell lysates were prepared by washing cells with cold PBS, followed by lysis buffer addition (50 mM Tris, 0.1 mM EGTA, 1 mM EDTA, 0.27 M sucrose, 1 % Triton X-100, Protease Inhibitor Cocktail (Roche) Phosphatase Inhibitor Cocktail (Sigma). Cell lysates were then collected and clarified by centrifugation at 12,500 rpm for 10 minutes at 4°C. Proteins were separated on Novex precasted SDS-PAGE Bis-Tris 4-12% gels (Invitrogen), using MES running buffer (Invitrogen). Proteins were then transferred to PVDF membranes (Millipore), blocked with 5 % milk and blotted with JAM2 (ThermoFisher PA5-21576) and GAPDH as loading control.

Animal studies

The mouse strain used in this study is $Jam2^{tm1.2Rha}$ for both *jam2* (encoding for Jam-B) knockout (KO) and wild-type (WT) mice. Mice were housed in the animal facility and were maintained on a 12-hour light dark cycle at a constant temperature and humidity. All animal experiments were carried out according to the UK Animal Act 1986 and approved by the UCL Animal Care Committee.

Generation of jam-2 deficient mice

The generation of this colony was previously described in the literature[5]. Briefly, a *jam2* (ortholog of human *JAM2*) gene fragment containing exon 5 was flanked by loxP recognition sites for Cre recombinase. Within the loxP-flanked region, a murine *jam2* cDNA fragment (codons 165-298, PEY…SFII*) followed by the bovine growth hormone polyadenylation signal (pA) was fused to a BspE1 site in exon 5. The construct also contained a neomycin resistance cassette surrounded by frt sites enabling Flp recombinase-mediated removal, as well as long (9 kb, 5′) and short (1.65 kb, 3′) arms for homologous recombination. Electroporated and G418-selected embryonic stem cell clones were analysed by Southern blot hybridization and polymerase chain reaction (PCR). For PCR screening, a primer pair, derived from exon 4 and the intronic *jam-2* sequence flanking the 5' loxP site, amplified a 345 bp band from WT chromosomes and a 450bp band from transgenic chromosomes (primers were 5′-AGACCGTGCTGAGATGATAGA-3′ and 5′-CCGAAGGAAGTGTCTAGTAAT-3′). Three independent lines were generated and maintained in a mixed 129Sv × C57BL/6 background. *jam-2* deficient mutants were generated by crossbreeding with the PGK-Cre line followed by interbreeding *jam2* KO/+ heterozygotes. Mice used in the present study were backcrossed for more than 12 generations on C57Bl/6 background.

Behavioural studies

Behavioural studies were performed in the *jam2* KO mice and compared to controls as described[6]. All mice were trained before the relevant tests. The balance beam walking test assesses mouse's ability to maintain balance while traversing a narrow beam to reach a safe platform by counting and comparing the number of missed steps between *jam2* KO and control mice. The footprint analysis assesses motor coordination and synchrony by examining gait during normal walking. The fore and hind paws were painted with dyes of different colours and the mice were encouraged to walk in a straight line over absorbent paper. Stance, stride

and sway measurements were compared among groups. A Mann-Whitney test (means ± SEM) was used to analyse the results of the behavioural tests.

Histological analysis

Mice were culled by injection of a pentobarbital overdose. The brains and the spinal cords were harvested and fixed in formalin. After formalin fixation the brains were cut sagittaly and the cervical, thoracic and lumbar spinal cord segments were cut transversely and processed for paraffin histology. The 5µm thin sections of the tissue were stained with haematoxylin and eosin (H&E) for routine examination. Representative sections from each brain and spinal cord were further assessed with Alcian Blue (Alcian Blue: Sigma, C174240; Gill III Haematoxylin: Thermo Shandon, 6765009, Schiffs: VWR, 351204L) and PAS (Ventana PAS Kit, Ventana 860-014) tinctorial stains and with immunohistochemical stains for Iba1 (Wako, 019-19741, 1:250), GFAP (Agilent, Z0334, 1:1000), NeuN (Chemicon, MAB377, 1:2000) and APP (Millipore, MAB348, 1:800). The Alcian Blue was hand-stained. The PAS tinctorial stain and immunostainings were performed on a ROCHE Ventana Discovery automated staining platform following the manufacturer's guidelines. Biotinylated secondary antibodies and a horseradish peroxidase-conjugated streptavidin complex and diaminobenzidine as a chromogen were used for the immunostainings. All staining was carried out with appropriate controls.

Automated quantification of histological results

For image analysis and automated pathology quantification, histological slides were first digitised on a LEICA SCN400F scanner (LEICA Milton Keynes, UK) at x40 magnification and 65% image compression setting during export. Then, from whole digitalised slides, the full thickness of cortex, entire midbrain and entire cerebellar white matter with cortical granule cell layer was manually selected for quantification studies. The percentage of neuropil vacuolation

on H&E stained sections, astrogliosis on GFAP immunostained sections and microglial activation on Iba1 immunostained slides in the manually selected cortical, midbrain and cerebellar regions was quantified, using an algorithm developed on open source digitalised image analysis software QuPath (v.0.2.0-m8)[7]. The difference of analysed parameters between wild-type and *jam2* KO mice in both young and old cohorts was analysed in Excel using paired t-test (with unequal variances), with $p < 0.05$ considered statistically significant.

Immunofluorescence analysis

Fibroblasts were fixed with 4% v/v formaldehyde in Dulbecco's Phosphate-Buffered Saline (DPBS) for 15 min at room temperature (RT). Slices of mouse brain were prepared as descried above for histological analysis. Cells/brain slices were incubated in blocking and permeabilisation solution (BPS) consisting of: 10% v/v donkey serum, 0.1% w/v triton-x100 in DPBS for 1 hr. Cells/slices were then incubated with primary antibodies diluted in BPS targeting JAM3/JAM-C (goat anti-JAM-C, RnD Systems AF1213, 10μg/ml), and TJP1 (Mouse anti-ZO-1, Thermofisher Scientific 339100, 5μg/ml) for 2 hrs. This was followed by incubation with donkey anti-goat Alex Fluor-488 (Thermofisher A11055, 1:1000) and donkey anti-mouse Alex Fluor-568 (Thermofisher a10037, 1:1000) fluorescently conjugated antibodies, and phalloidin-647 for 1 hr. Nuclei were counterstained with 0.1 μg/ml 4′,6-diamidino-2phenylindole (DAPI) and mounted with mounting medium (Dako) for confocal microscopy. Images were acquired with a Zeiss 710 VIS CLSM equipped with a META detection system using 20x and 63x (oil immersion) objectives, and max intensity projections prepared using ImageJ FIJI software.

References

1. Seelow, D., Schuelke, M., Hildebrandt, F., and Nürnberg, P. (2009). HomozygosityMapper--an interactive approach to homozygosity mapping. Nucleic Acids Res. *37*, W593-599.

2. Wang, K., Li, M., and Hakonarson, H. (2010). ANNOVAR: functional annotation of genetic variants from high-throughput sequencing data. Nucleic Acids Res. *38*, e164.

3. Retterer, K., Juusola, J., Cho, M.T., Vitazka, P., Millan, F., Gibellini, F., Vertino-Bell, A., Smaoui, N., Neidich, J., Monaghan, K.G., et al. (2016). Clinical application of whole-exome sequencing across clinical indications. Genet. Med. *18*, 696–704.

4. Bauer, P., Kandaswamy, K.K., Weiss, M.E.R., Paknia, O., Werber, M., Bertoli-Avella, A.M., Yüksel, Z., Bochinska, M., Oprea, G.E., Kishore, S., et al. (2019). Development of an evidence-based algorithm that optimizes sensitivity and specificity in ES-based diagnostics of a clinically heterogeneous patient population. Genet. Med. *21*, 53–61.

5. Arcangeli, M.-L., Frontera, V., Bardin, F., Obrados, E., Adams, S., Chabannon, C., Schiff, C., Mancini, S.J.C., Adams, R.H., and Aurrand-Lions, M. (2011). JAM-B regulates maintenance of hematopoietic stem cells in the bone marrow. Blood *118*, 4609–4619.

6. Brooks, S.P., and Dunnett, S.B. (2009). Tests to assess motor phenotype in mice: a user's guide. Nat. Rev. Neurosci. *10*, 519–529.

7. Bankhead, P., Loughrey, M.B., Fernández, J.A., Dombrowski, Y., McArt, D.G., Dunne, P.D., McQuaid, S., Gray, R.T., Murray, L.J., Coleman, H.G., et al. (2017). QuPath: Open source software for digital pathology image analysis. Sci Rep *7*, 16878.
