# Supplementary material for: Bi-allelic JAM2 Variants Lead to Early-Onset Recessive Primary Familial Brain Calcification
Source: Am J Hum Genet. 2020 Mar 5;106(3):412–21. doi: 10.1016/j.ajhg.2020.02.007 (PMC7058839; doi:10.1016/j.ajhg.2020.02.007)
Supplement: Document S2. Article plus Supplemental Information [file mmc4.pdf]

REPORT

Bi-allelic *JAM2* Variants Lead to Early-Onset Recessive Primary Familial Brain Calcification

Lucia V. Schottlaender,[1,2,3] Rosella Abeti,[4] Zane Jaunmuktane,[4,5] Carol Macmillan,[6] Viorica Chelban,[1] Benjamin O'Callaghan,[1] John McKinley,[7,8] Reza Maroofian,[1] Stephanie Efthymiou,[1] Alkyoni Athanasiou-Fragkouli,[1] Raeburn Forbes,[9] Marc P.M. Soutar,[10] John H. Livingston,[11] Bernardett Kalmar,[1] Orlando Swayne,[4,12] Gary Hotton,[4,12] SYNAPS Study Group,[1] Alan Pittman,[1] João Ricardo Mendes de Oliveira,[13] Maria de Grandis,[14] Angela Richard-Loendt,[10] Francesca Launchbury,[10] Juri Althonayan,[4] Gavin McDonnell,[8] Aisling Carr,[1,12] Suliman Khan,[15] Christian Beetz,[15] Atil Bisgin,[16] Sevcan Tug Bozdogan,[16] Amber Begtrup,[17] Erin Torti,[17] Linda Greensmith,[1] Paola Giunti,[4] Patrick J. Morrison,[18] Sebastian Brandner,[5] Michel Aurrand-Lions,[14] and Henry Houlden[1,12,19,*]

Primary familial brain calcification (PFBC) is a rare neurodegenerative disorder characterized by a combination of neurological, psychiatric, and cognitive decline associated with calcium deposition on brain imaging. To date, mutations in five genes have been linked to PFBC. However, more than 50% of individuals affected by PFBC have no molecular diagnosis. We report four unrelated families presenting with initial learning difficulties and seizures and later psychiatric symptoms, cerebellar ataxia, extrapyramidal signs, and extensive calcifications on brain imaging. Through a combination of homozygosity mapping and exome sequencing, we mapped this phenotype to chromosome 21q21.3 and identified bi-allelic variants in *JAM2*. *JAM2* encodes for the junctional-adhesion-molecule-2, a key tight-junction protein in blood-brain-barrier permeability. We show that *JAM2* variants lead to reduction of *JAM2* mRNA expression and absence of JAM2 protein in patient's fibroblasts, consistent with a loss-of-function mechanism. We show that the human phenotype is replicated in the *jam2* complete knockout mouse (*jam2* KO). Furthermore, neuropathology of *jam2* KO mouse showed prominent vacuolation in the cerebral cortex, thalamus, and cerebellum and particularly widespread vacuolation in the midbrain with reactive astrogliosis and neuronal density reduction. The regions of the human brain affected on neuroimaging are similar to the affected brain areas in the *myorg* PFBC null mouse. Along with *JAM3* and *OCLN*, *JAM2* is the third tight-junction gene in which bi-allelic variants are associated with brain calcification, suggesting that defective cell-to-cell adhesion and dysfunction of the movement of solutes through the paracellular spaces in the neurovascular unit is a key mechanism in CNS calcification.

Primary familial brain calcification (PFBC [MIM: 213600]), often referred as Fahr disease, constitutes a heterogeneous neurodegenerative disorder that presents with mineral calcium deposits in the brain. The clinical manifestations can include, but are not restricted to, movement disorders such as parkinsonism and ataxia, seizures, migraine, and neuropsychiatric symptoms. Both autosomal-dominant and -recessive inheritance patterns have been reported.[1] The clinical picture and severity are often variable between and within families, with some family members being clinically asymptomatic. The advent of widespread brain imaging for individuals presenting with parkinsonism, who were previously clinically diagnosed, has significantly increased the number of PFBC-affected families identified.[2]

There are two main pathogenic mechanisms described so far in PFBC. On the one hand, the calcium and phosphate homeostasis dysfunction via dominant mutations in *SLC20A2* (MIM: 158378) and *XPR1* (MIM: 605237).[1] The inhibition of phosphate uptake by mutations in *SLC20A2* encoding for sodium-dependent phosphate transporter 2 (PiT-2) leads to deposition of calcium in the vascular extracellular matrix, and inhibition of phosphate export associated with *XPR1* mutations is expected to

[1]Department of Neuromuscular Diseases, UCL Queen Square Institute of Neurology, Queen Square, WC1N3BG London, UK; [2]Dubowitz Neuromuscular Centre, UCL Great Ormond Street Institute of Child Health, WC1N 1EH London, UK; [3]Argentine National Scientific and Technological Research Council (CONICET), C1425FQB Buenos Aires, Argentina; FLENI Neurological Research Institute, C1428 AQK Buenos Aires, Argentina; [4]Department of Clinical and Movement Neurosciences, UCL Queen Square Institute of Neurology, WC1N3BG London, UK; [5]Division of Neuropathology, The National Hospital for Neurology and Neurosurgery, University College London Hospitals NHS Foundation Trust, Queen Square, London WC1N 3BG, UK; [6]Department of Pediatrics, University of Chicago, Chicago, IL 60637, USA; [7]Department of Neurology, Dublin Neurological Institute at the Mater Misericordiae University Hospital, 57 Eccles St, Dublin 7 DO7W7XF, Ireland; [8]Regional Neurosciences Centre, Royal Victoria Hospital, Belfast BT12 6BA, UK; [9]Neurology Centre, Southern HSC Trust, Craigavon Area Hospital, Portadown BT63 5QQ, UK; [10]Department of Neurodegenerative Disease, UCL Queen Square Institute of Neurology, Queen Square, London WC1N 3BG, UK; [11]Paediatric Neurology, The Leeds Teaching Hospitals NHS Trust, Leeds General Infirmary, Leeds LS1 3EX, UK; [12]The National Hospital for Neurology and Neurosurgery, Queen Square, London WC1N 3BG, UK; [13]Universidade Federal de Pernambuco, Departamento de Neuropsiquiatria, Recife 50670-901, Brazil; [14]Aix-Marseille University, Inserm, CNRS, Institut Paoli-Calmettes, CRCM, 13009 Marseille, France; [15]CENTOGENE AG, Rostock 18055, Germany; [16]Medical Genetics Department of Medical Faculty & AGENTEM (Adana Genetic Diseases Diagnosis and Treatment Center), Çukurova University, Adana 01330, Turkey; [17]GeneDx, 207 Perry Parkway, Gaithersburg, MD 20877, USA; [18]Centre for Cancer Research and Cell Biology, Queens University, Belfast BT9 7AE, UK; [19]Neurogenetics Laboratory and Clinical Service, The National Hospital for Neurology and Neurosurgery, Queen Square, London WC1N 3BG, UK
*Correspondence: h.houlden@ucl.ac.uk
https://doi.org/10.1016/j.ajhg.2020.02.007.
© 2020 The Authors. This is an open access article under the CC BY license (http://creativecommons.org/licenses/by/4.0/).

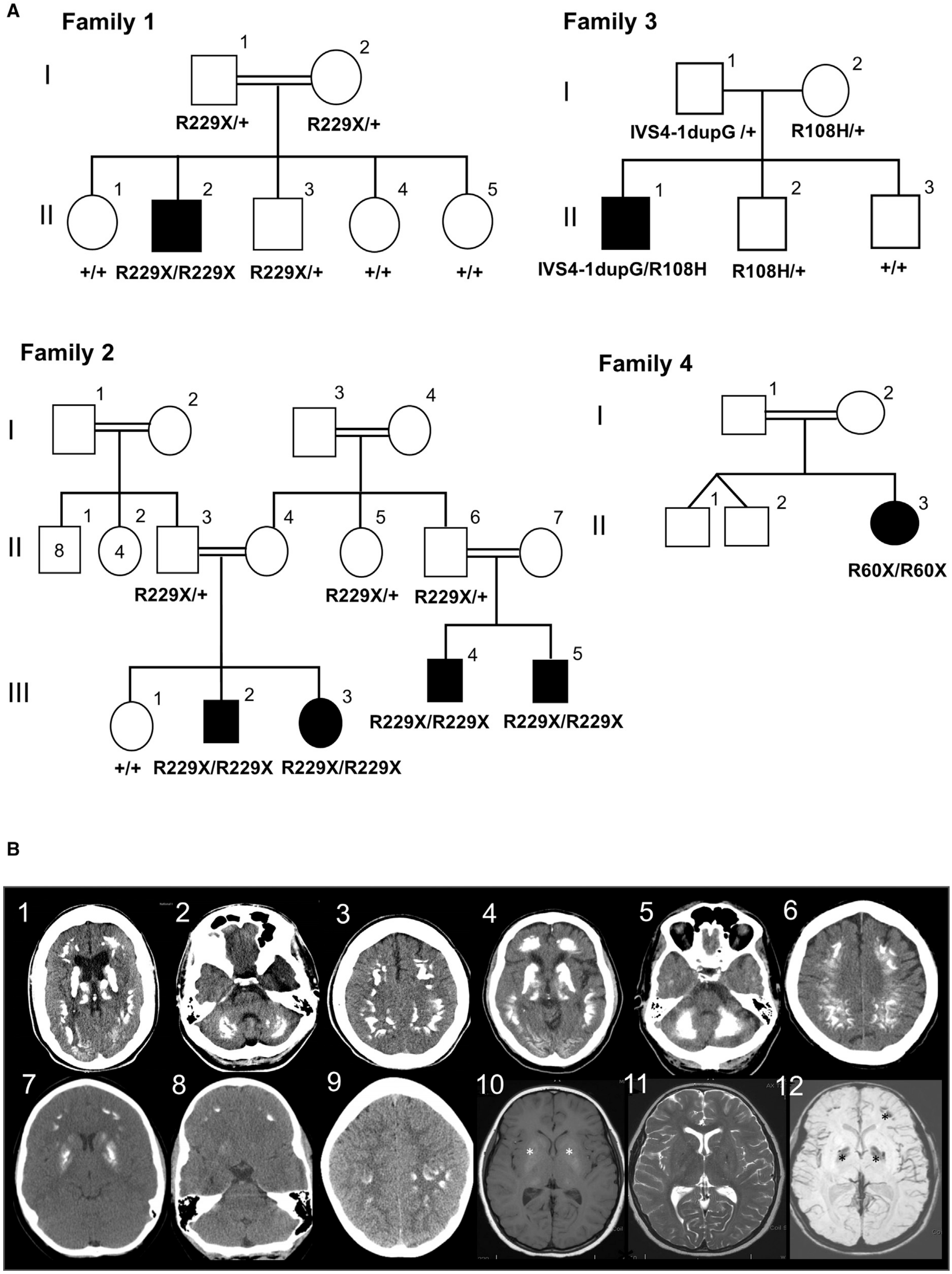

Figure 1. Clinical and Neuroimaging Features of *JAM2*-Related Disease
(A) Pedigrees of the four families with bi-allelic *JAM2* mutations.
(B) Brain images of *JAM2*-related disease. B1 to B3 are CT scans acquired from case subject F1-II:2 from family 1 and B4 to B6 are CT acquired from case subject F2-III:3 from family 2. In both individuals there is extensive, symmetrical, bilateral calcification involving the

(legend continued on next page)

increase intracellular phosphate concentration and provoke calcium phosphate precipitation.[3]

On the other hand, the second mechanism causes PFBC through disruption of the neurovascular unit (NVU). Endothelial integrity and function affecting the blood-brain barrier (BBB) is altered via dominant mutations in *PDGFB* (MIM: 190040) and *PDGFRB* (MIM: 173410) encoding for the platelet-derived growth factor B and its receptor, that lead to the impairment of pericytes recruitment and BBB integrity, causing vascular and perivascular calcium accumulation.[2] The recessive brain calcification phenotype due to *MYORG*[4] (MIM: 618255) mutations, has been shown to present specific *myorg* mRNA expression in mouse astrocytes and disturb the normal function of the NVU. Mutations in *JAM3*[5] (MIM: 613730) and *OCLN*[6] (MIM: 602876) encoding for tight junction proteins lead to excess solutes crossing the BBB causing CNS calcification and hemorrhage.

Interestingly, a study on the *Slc20a2* null mouse suggested that the calcified nodules present in the brain initiated in pericytes and astrocytes and found endogenous IgG around nodules proposing that there was increased BBB permeability[7] and identifying a possible link between different PFBC causative genes.

Pathologically, human brains exhibit calcium salt deposits predominantly distributed around small blood vessels,[2] and a reported subject with a *SLC20A2* mutation presented calcification in the tunica media of small arteries, arterioles, and capillaries, but not in veins distributed in the basal ganglia, thalamus, cerebellar white matter, and deeper layers of the cerebral cortex.[8]

Despite important progress in discovering the genetic architecture of PFBC, more than half of the case subjects remain genetically unsolved.[2]

In this study we report four unrelated families with seven individuals affected by autosomal-recessive primary familial brain calcification. In the families described here, we used a combination of homozygosity mapping, exome sequencing (ES), functional studies, and mouse model to identify and characterize the causal variants in *JAM2* (MIM: 606870) encoding for the junctional-adhesion-molecule-2, a tight-junction protein as a cause of PFBC.

Two unrelated consanguineous families from traveller communities in England (family 1) and Northern Ireland (family 2), one non-consanguineous family from the United States (family 3) identified using GeneMatcher, and one Turkish consanguineous family (family 4) were included in this study (Figure 1A). Clinical features of affected individuals are presented in Table 1. The proband in family 1 (F1-II:2) had a normal birth and early milestones. He presented with childhood-onset cerebellar ataxia and learning difficulties. His symptoms progressed and were associated with additional behavioral problems and worsening cognitive impairment. He was examined by a neurologist at the age of 23 years old. At that stage, he already had difficulties following commands and had alternate exotropia with left eye preference for fixation, slow and jerky pursuit, and ophthalmoplegia. He had reduced ability to control tongue movements and was unable to protrude his tongue. He was dysarthric with dysphagia and a percutaneous endoscopic gastrostomy (PEG) insertion at the age of 22 years. He had increased tone in the upper and lower limbs with ankle contractures, brisk reflexes throughout, and upgoing plantars. There was upper and lower limb ataxia, bradykinesia, and generalized dystonia, worse in the upper limbs (Video S1). Occasional seizures were seen later in the disease course. The interictal electroencephalography (EEG) showed moderate generalized slowing of cortical rhythms.

There were four affected individuals in the second family. Two siblings (F2-III:2 and F2-III:3) presented with borderline low IQ in childhood but had no definite physical limitations in early life. In their twenties they had social withdrawal and severe depression requiring treatment. The disease progressed and examination at 41 and 39 years revealed severe speech hypophonia, dysphagia, hypomimia, reduced vertical up gaze, orofacial dyskinesias, slow and reduced tongue movements with bradykinesia, and dystonic limb posturing in both affected individuals. Tone was increased in an extrapyramidal pattern with lower limb hyperreflexia and extensor plantar responses, grasp reflexes, positive glabellar tap, and brisk jaw jerk. They both had memory decline with impaired recall. Treatment with ropinirole did not lead to any significant improvement in symptoms. The disease progressed, they became bedridden and case F2-III:2 needed a PEG insertion 10 years after the onset of movement problems due to recurrent aspiration pneumonia, and the proband died in the late 40s. Case subjects F2-III:4 and F2-III:5 are maternal first cousins of the index case subject in family 2. They presented an almost identical phenotype. On a background of depression, both brothers noted progressive "slurring" of speech, slowing of all movements, difficulty with walking, recurrent falls, and poor memory. Examination demonstrated a similar phenotype with dysarthria, abnormal pursuit with frequent saccadic intrusions, pronounced bradykinesia, extrapyramidal rigidity, and bilaterally extensor plantar responses.

The proband in family 3 (F3-II:1) had normal early development but later developed mild delay in fine motor and language milestones that were progressive. He also developed mild coordination problems and autism spectrum disorder (ASD) and received special education services at school age. At age 11 years, repeat neuropsychologic

basal ganglia, deep cortical gray matter and cerebellum. B7, 8, and 9 are CT from case subject F3-II:1 demonstrating calcification in the basal ganglia and cortical gyri but not in the cerebellum. B10, 11, and 12 are axial MRI scans from individual F3-II:1 from family 3 showing basal ganglia and frontal calcification (B10 is T1 MRI, B11 is T2 MRI, and B12 is Axial Ven Bold reconstruction MRI that is sensitive to calcium shown as hypointense regions). Asterisk (*) areas of calcification on MRI.

Table 1. Clinical Features of Affected Individuals with *JAM2* Bi-allelic Variants

	Individual F1-II:2	Individual F2-III:2	Individual F2-III:3	Individual F2-III:4	Individual F2-III:5	Individual F3-II:1	Individual F4-II:3
cDNA sequence	c.685C>T	c.685C>T	c.685C>T	c.685C>T	c.685C>T	c.395−1dupG, c.323G>A	c.177_180delCAGA
Amino acid change	p.Arg229Ter	p.Arg229Ter	p.Arg229Ter	p.Arg229Ter	p.Arg229Ter	IVS4-1dupG, p.Arg108His	p.Arg60Ter
Zygosity	homozygous	homozygous	homozygous	homozygous	homozygous	compound heterozygous	homozygous
Gender (male/female)	male	male	female	male	male	male	female
Birth and early milestones	normal	normal	normal	normal	normal	normal	normal
Onset of symptoms	childhood	late 20s	late 30s	teenage	teenage	childhood	early childhood
Symptom at onset	cerebellar ataxia and cognitive decline	cognitive decline, depression	difficulty walking	depression, dysarthria	depression, dysarthria	autism spectrum disorder	seizures
Age at examination (in years)	24	41	39	40	49	15	7
**Phenotype at Last Examination**							
Pyramidal syndrome	yes; increased tone, brisk reflexes, upgoing plantars	yes; increased tone, brisk reflexes, upgoing plantars	yes; increased tone, brisk reflexes, upgoing plantars	yes; increased tone, brisk reflexes, upgoing plantars	yes; increased tone, brisk reflexes, upgoing plantars	no	no
Cerebellar syndrome	yes; upper and lower limb ataxia, dysarthria, nystagmus	yes; upper and lower limb ataxia	yes; upper and lower limb ataxia	yes; upper and lower limb ataxia, dysarthria	yes; upper and lower limb ataxia, dysarthria	yes; upper and lower limb mild ataxia, nystagmus	no
Parkinsonism	yes; rigidity, bradykinesia.	yes; hypophonia, hypomimia, bradykinesia	yes; hypophonia, hypomimia, bradykinesia	yes; rigidity, bradykinesia	yes; rigidity, bradykinesia	no	no
Dystonia	yes; generalized	yes; limb dystonia and orofacial dyskinesias	yes; limb dystonia and orofacial dyskinesias	no	no	no	no
Other	seizures, ophthalmoplegia, PEG inserted in advance stage	PEG inserted in advance stage	became anarthric in advanced stage	–	–	autism spectrum disorder	–
Cognitive function	severe cognitive decline	memory decline with severe impaired recall	unable to comment on cognition due to anarthria.	severe cognitive decline	severe cognitive decline	decline in academic performance	normal for her age
Brain imaging calcification pattern	basal ganglia, thalamus, cerebellum, deep gray matter	basal ganglia, thalamus, cerebellum, deep gray matter	basal ganglia, thalamus, cerebellum, deep gray matter	basal ganglia, thalamus, cerebellum, deep gray matter	basal ganglia, thalamus, cerebellum, deep gray matter	basal ganglia, and frontal cortex	basal ganglia, dentate nucleus and cerebellar hemispheres

evaluation showed a continuous decline in academic performance. Parents were asymptomatic, an older brother had some anxiety and hyperactivity, and a younger brother aged 13 years had mild autistic features. At the last examination of the proband aged 15 years, he had autism spectrum features, hyperactivity, developmental delay, and coordination problems. The coordination difficulties were mild but affected both fine motor skills (buttons, zippers, hooks) and complex gross motor tasks. His learning difficulties were progressive.

The affected member of family 4 (F4-II:3) was born to Turkish consanguineous parents and presented with seizures when she was 18 months of age; current age is 7.5 years and she has had a total of 3 seizures. Her development so far has been in keeping with her peers and her last examination did not reveal any neurological signs. Brain calcification was identified on MRI imaging with bilateral symmetric calcification of the basal ganglia, dentate nucleus, and subcortical white matter of cerebellar hemispheres. Prior to exome sequencing, the known Fahr's genes was sequenced and negative.

All seven case subjects reported here had brain calcification identified on brain CT and/or MRI imaging. They had a consistent pattern of bilateral symmetric calcification of the basal ganglia and deep cortical gray matter. In addition, the older individuals from families 1 and 2 had severe calcification in the cerebellum folia and the thalamus (Figure 1B). All families had extensive genetic, metabolic, and mitochondrial investigations carried out that excluded acquired and other inherited causes of brain calcification.

In order to localize the chromosomal location of the pathogenic variant, we genotyped three affected and two unaffected individuals from extended family 2 genome-wide by using Illumina HumanCytoSNP-12v2-1 Beadchip array incorporating ~200,000 genetic markers. Three regions of homozygosity were detected on chr10:37,414,883–43,132,376, chr13:88,327,643–93,518,692, and chr21:22,370,881–28,338,710. Next, we performed exome sequencing on probands of families 1 and 2 to identify the causative variant(s). On the assumption that the disease follows an autosomal-recessive pattern of inheritance in the families as well as presence of consanguinity in two families, we prioritized the bi-allelic potentially functional variants residing within the runs of homozygosity. These variants were screened through all publicly available population databases and our in-house database. We excluded synonymous variants, intronic variants (>7 bp from exon boundaries) and common variants (minor allele frequency > 0.001%). The selected variants were validated, and segregation analysis was performed using Sanger sequencing.

In families 1 and 2, filtered exome-sequencing data narrowed down the variants to the same homozygous nonsense variant (GenBank: NM_021219; c.685C>T [p.Arg229Ter]) in *JAM2* residing within a 5 Mb region of homozygosity on chr21q21.3 (Figure S1). Sanger sequencing verified the correct segregation of the variant on available samples of both families (Figures 1A, 2A, and 2B). This variant was absent in our in-house exome database of more than 10,000 exomes, absent in homozygous state in all publicly available databases, and present in heterozygous state with a minor allele frequency (MAF) of 0.00002 in gnomAD (6/280662). The early stop codon introduced by c.685C>T is predicted to result in production of a truncated protein lacking the transmembrane and cytoplasmic regions of *JAM2* and cause reduction in total *JAM2* as a consequence of nonsense-mediated decay (NMD) of the mutant transcript. Indeed, RT-PCR analysis confirmed a reduction of *JAM2* mRNA expression levels in the proband in family 1 compared to the heterozygous carrier and unrelated control subject (Figure 2C). Furthermore, western blot analysis confirmed the absence of JAM2 protein in the homozygous proband (Figure 2D). The reduction of RNA expression and absent JAM2 protein in the fibroblast cell lines of the proband support the loss-of-function role of this variant. As JAM2, JAM3, and TJP1 proteins are all junctional components and associated with brain calcification phenotype, we investigated whether *JAM2* c.685C>T variant affected the localization of the other two proteins. We show that there was no difference in the localization of JAM3 and TJP1 in primary dermal fibroblasts from *JAM2* homozygous affected individuals (Figure S2).

In family 3, Trio-ES revealed compound heterozygous variants in *JAM2* (GenBank: NM_021219; c.395−1dupG [IVS4-1dupG] and c.323G>A [p.Arg108His]). The c.395−1dupG was inherited from the father and c.323G>A was inherited from the mother. The c.323G>A was reported once in gnomAD in heterozygous state (MAF 0.00003, 1/31408), three times in GeneDx in-house database (MAF 0.00002, 3/167854), and was absent in all databases as homozygous. It disrupts a highly conserved residue (CADD:34) and is predicted to be damaging to the protein function by *in silico* prediction tools (SIFT, PROVEAN, Mutation Taster, Mutation Assessor, and PolyPhen). The c.395−1dupG is predicted to cause the retention of the canonical splice acceptor site of intron 4 with insertion of the G nucleotide as the first base of exon 5 causing a frameshift and a truncated protein (p.Val132GlyfsX9). This variant was absent from all public databases and present five times in heterozygous state in GeneDx in-house database (MAF 0.00003, 5/171284). The variants segregated fully within the family (Figures 1A, 2A, and 2B).

In family 4, clinical ES uncovered a homozygous 4 bp deletion in *JAM2* (GenBank: NM_021219; c.177_180delCAGA [p.Arg60Ter]) (Figures 1A, 2A, and 2B) that causes a frameshift and an early termination of the protein affecting the extracellular, transmembrane, and cytoplasmic domains of JAM2. The variant was not reported in any public databases and was predicted pathogenic by MutationTaster causing loss of function by NMD.

In order to characterize the link between *JAM2* variants and the human neurological phenotype, we developed

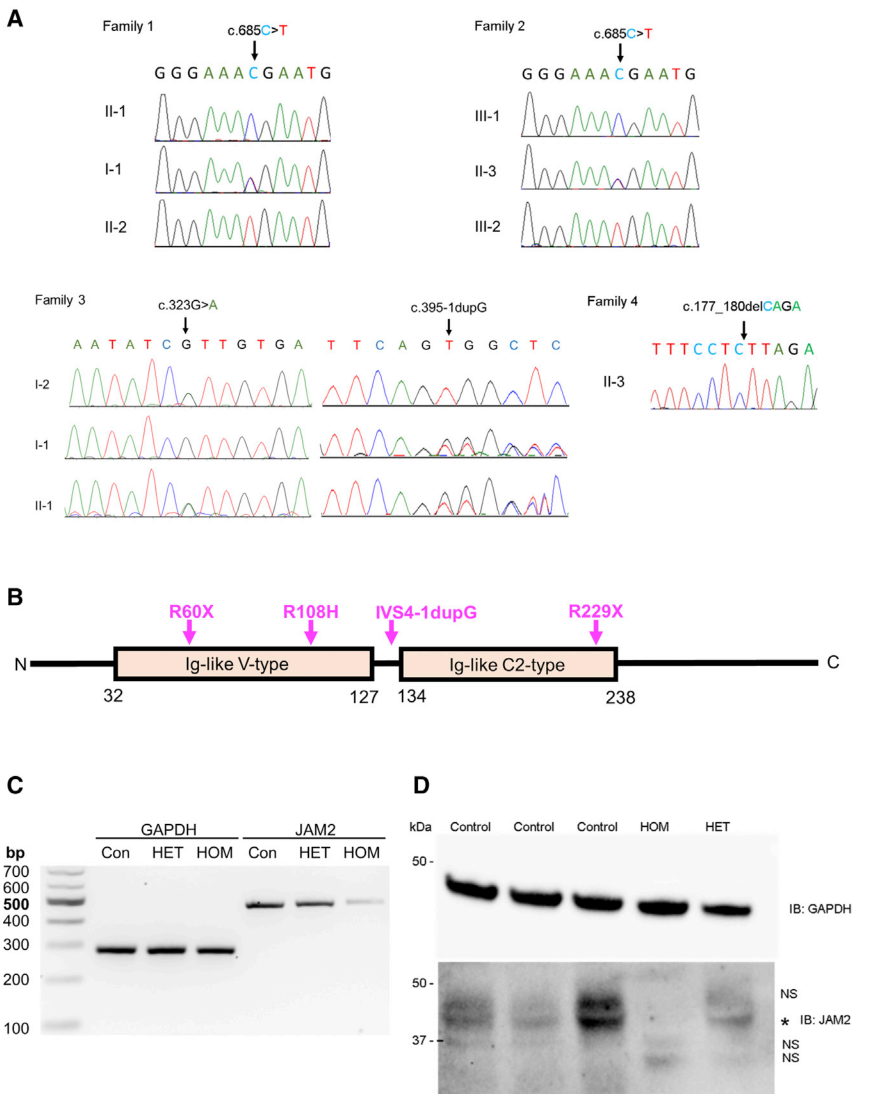

Figure 2. Validation of *JAM2* Variants Identified in This Study

(A) Validation by Sanger sequencing of the stop variant c.685C>T (p.Arg229Ter) in families 1 and 2, the bi-allelic variants in family 3 (IVS4-1dupG and p.Arg108His), and the homozygous change in family 4 c.177_180delCAGA (p.Arg60Ter).

(B) Protein structure of JAM2 showing the 2 main domains (Ig-like V-type and Ig-like C2-type) as well as the variants identified in the 4 families.

(C) Total RNA and protein extracts were prepared from fibroblast cell lines isolated from the proband of family 1 (HOM), his unaffected mother (HET), and unrelated control subjects (Con) to assess the c.685C>T (p.Arg229Ter) *JAM2* variant. Agarose gel separation of RT-PCR products shows a reduction in *JAM2* mRNA expression in the homozygous proband (HOM) compared to the proband's mother (HET) and unrelated control subject (Con). *GAPDH* RT-PCR served as a loading control.

(D) Western blot analysis of total protein lysates using anti-JAM2 and GAPDH (loading control) antibodies revealed presence of JAM2 protein in control (Control) and unaffected carrier (HET) cells but complete loss of JAM2 protein in the homozygous proband of family 1 (HOM). NS, non-specific band; asterisk, JAM2.

jam2 knockout (*jam2* KO) mice. Behavioral tests in the *jam2* KO mice showed significant difficulties in beam walking test and gait abnormalities when compared to wild-type mice (Figure 3). There was a significant reduction in stride length (wild-type: 8.14 ± 0.9, *jam2* KO: 6.3 ± 1; ***$p < 0.0001$) and increase in sway length (wild-type: 0.13 ± 0.14, *jam2* KO: 0.9 ± 0.4; **$p = 0.002$) when comparing *jam2* KO to wild-type littermates' controls (Figure 3A). Additionally, the number of missed steps (wild-type: 1.2 ± 1.3, *jam2* KO: 6.5 ± 3.6; *$p = 0.017$) in the beam-walking test was higher in *jam2* KO compared to controls (Figure 3B and 3C, Video S2).

Brains of two *jam2* KO and two wild-type (C57BL/6) mice were examined at a young age (6 months old) and four *jam2* KO and four wild-type mice were examined at an old age (18 months old). In addition, spinal cord sections from one of the young *jam2* KO mice and from all old *jam2* KO and control mice were examined. We observed prominent widespread vacuolation in the midbrain and some in the thalamus and cerebral and cerebellar cortex of young *jam2* KO mice. In the midbrain, the vacuolar change was accompanied by prominent reactive astrogliosis, mild microglial activation, and mild reduction in the neuronal density compared to controls (Figure 4). Brains of aged *jam2* KO mice showed similar changes, with prominent widespread neuropil vacuolation in the midbrain accompanied by marked astrogliosis, mild microglial activation, and moderately reduced neuronal density. In contrast to young *jam2* KO mice, there was more prominent vacuolation in the cerebral cortex, thalamus, and cerebellar cortex and particularly widespread vacuolation in the cerebellar white matter. To a lesser extent, neuropil vacuolation in the same regions was also seen in the age-matched control wild-type mice, suggesting that *jam2* KO mice develop age-related changes at a much younger age, which in some areas, such as cerebellar white matter, midbrain, thalamus, and cerebellar cortex, increase in severity with age. In addition, we performed automated quantification of the neuropil vacuolation on H&E-stained sections, GFAP immunoreactive gliosis and Iba1-positive microglial activation in young and aged wild-type and *jam2* KO mice (Figure S3). Automated quantification of the percentage of vacuolation, gliosis, and microglial activation in the cortex, midbrain, and cerebellum was performed on digitalized slides, using open source software

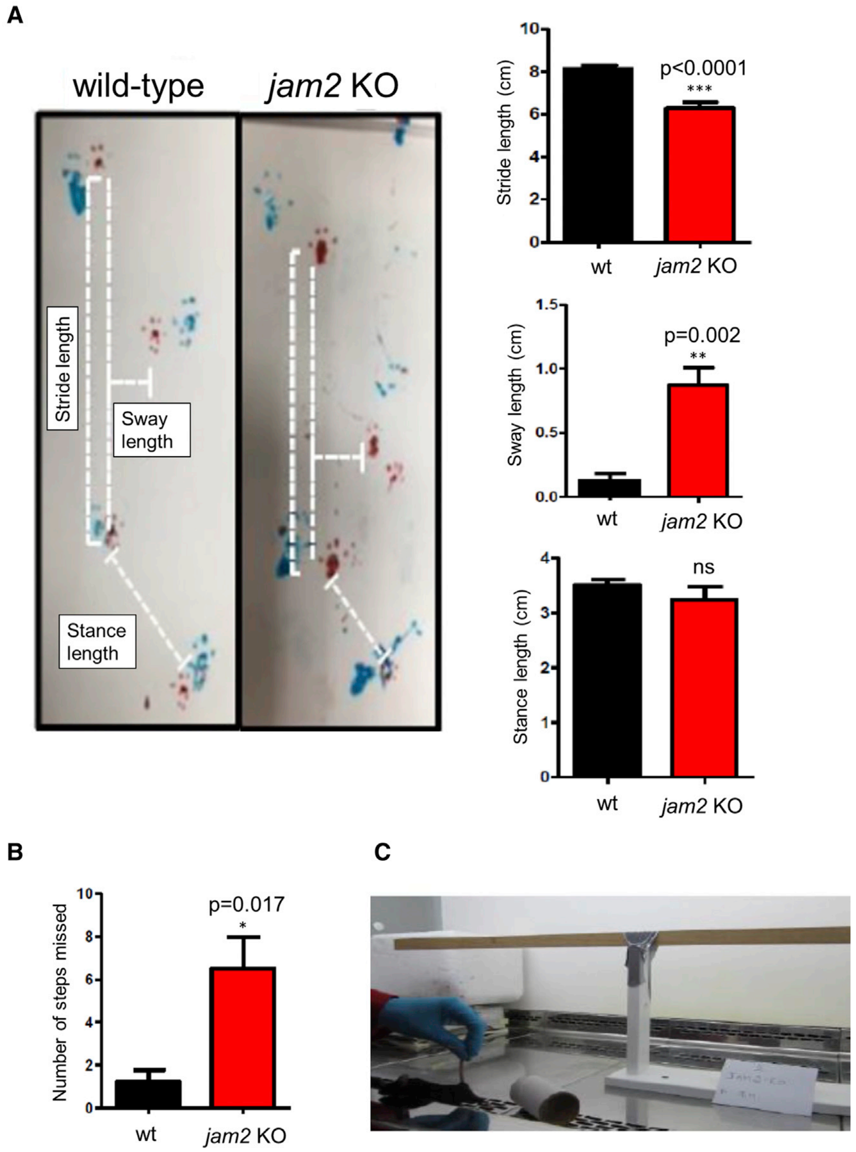

Figure 3. Behavioral Study on *jam2* KO Mice and Wild-Type (WT)
(A) Representative images of wild-type (left) and *jam2* KO strides (right) in the gait test. Forepaw (blue) and hindpaw (red). Altered gait of the *jam2* KO mice was analyzed in stance length, stride, and sway compared to wild-type (***$p < 0.0001$, **$p = 0.002$; means ± SEM; $n = 6$ mice per genotype).
(B and C) Walking beam performance on test day, showing elevated latency to cross the beam in *jam2* KO (*$p = 0.017$; means ± SEM; $n = 5$ wild-type, $n = 6$ *jam2* KO). See Video S2 for example beam walking test of a *jam2* KO mouse and a control (wild-type).

QuPath. There was a significant increase in the degree of neuropil vacuolation ($p < 0.00007$) and astrogliosis ($p < 0.0138$) in the midbrain of old *jam2* KO mice when compared with age-matched wild-type mice, whereas Iba1-positive microglial activation in old *jam2* KO mice was less pronounced than in age-matched wild-type mice ($p < 0.035$). No mineralization or calcification was observed in the brains of young or old *jam2* KO mice or controls at the time of examination.

The spinal cord morphology was similar in young and old *jam2* KO-deficient mice but remarkably differed from that of control mice. The main findings in *jam2* KO spinal cords were widespread neuronal, perivascular, and neuropil mineralization as well as widespread vacuolation in the gray matter. The mineralized deposits were negative for PAS and showed weak reactivity for Alcian blue, excluding calcification, cartilagination, or ossification stage. The mineralization and vacuolar change were evident across the gray matter in both anterior and posterior horns bilaterally and at all levels of the spinal cord. Although mineralization and calcification are known to occur with age in wild-type mice, in the four spinal cords from our control group (age-matched to old *jam2* KO mice), no mineralization or neuropil vacuolation at any of the spinal cord levels was observed (Figure S4). JAM3 and TJP1 tight-junction proteins were investigated in the KO mice, and there was no difference in localization between affected and wild-type mice, in different areas of the brain (Figures S5 and S6).

Mouse models of other brain calcification genes have shown brain calcification in similar areas to those found in humans. Such is the case of *Slc20a2*-null mice exhibiting progressive calcification of the thalamus, basal ganglia, and cortex beginning at 8 weeks and present in close proximity or within blood vessels and affecting astrocytes and perycites[7,9,10] and for *myorg*-null mice developing calcifications at 9 months.[4] A study on mice carrying hypomorphic *Pdgfb* alleles interestingly showed that the calcifications depend on the loss of endothelial PDGF-B and correlate with the degree of pericyte and BBB deficiency.[11] *Ocln*-null mice present with similar calcification surrounding blood vessels as seen in an autopsied case,[6] but there were no abnormalities reported in the brain of *jam3* KO mice, and there was absence of Jam3 in the vasculature of the adult mouse brain.[5] In our study, we were not able to detect calcification in the brain of our *jam2* mouse model even though we studied these mice until the age of 18 months, an age at which other PFBC models already present calcification and thus it does not seem to be age related. However, our *jam2* KO mice did show prominent vacuolation, inflammatory response, and neuronal density reduction in the same areas affected by calcification in humans. Further studies are needed to

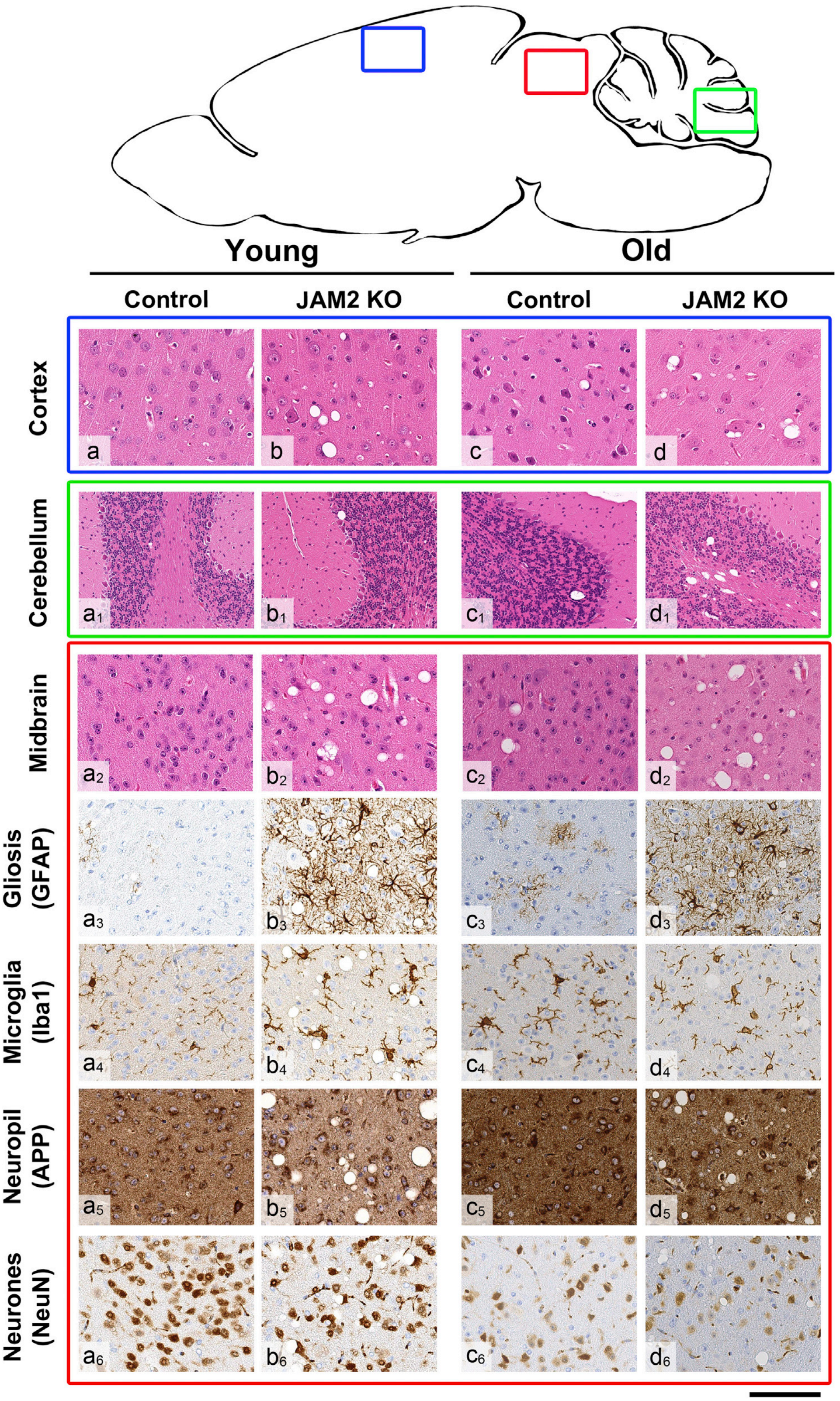

Figure 4. Brain Pathology in Control and *jam2* KO Mice of Young (6 Months Old) and Old Age (18 Months Old)
Littermate controls (wild-type) (a, a1–a6) show no significant pathology on (H&E) stained sections (a, cortex; a1, cerebellum; a2, midbrain) and on immunohistochemistry for astrocytes (GFAP) (a3, midbrain), microglia (Iba1) (a4, midbrain), neuropil (APP) (a5, midbrain), and neurones (NeuN) (a6, midbrain). In age-matched *jam2* KO mice, occasional cortical vacuolation is evident in the cerebral (b) and cerebellar (b1) cortex and widespread prominent vacuolation is seen in the midbrain (b2). In the midbrain region there is also marked reactive astrocytosis (b3) and mild microglial activation (b4). APP immunostaining highlights vacuolar change in the neuropil (b5) and NeuN shows mild reduction in the neuronal density (b6). Aged wild-type mice show occasional vacuolation in the cerebral

(legend continued on next page)

understand better the disease mechanisms and the differences between humans and mice for *JAM2* and also *JAM3*.

Together, our results confirm that *JAM2* variants underlie the phenotype in the four families reported here. This implicates *JAM2* as a cause of human Mendelian disease. *JAM2* encodes for the junctional-adhesion-molecule-2 (JAM2), member of the junctional adhesion molecules family, localized in the tight junctions of endothelial cells and the NVU.[12,13] JAM2 is an adhesive ligand interacting with various immune cell types and regulating vascular function and was recently identified as an inhibitor of somatodendritic myelination in spinal cord neurons.[14]

Junctional adhesion molecules are a family of proteins that play an important role in the regulation of cell polarity, endothelium permeability, and leukocyte migration and the blood-brain-barier (BBB) function. In addition to *JAM2*, recessive variants in *JAM3* and *OCLN* were linked to complex neurological disorders presenting with calcification in the brain,[5,6] suggesting that deregulation of the central NVU is important in pathogenesis of PFBC. Even though increased permeability of the BBB hasn't been confirmed in *jam2* KO mice so far,[13] including this study, mutations have now been identified in three genes encoding tight junction proteins in humans (*JAM2*, *JAM3*, and *OCLN*), suggesting that loss of cell-to-cell adhesion with subsequent dysfunction of solute passage is an important cause of brain calcification. Furthermore, a multicenter collaboration from China recently described four patients from three families that presented with PFBC and bi-allelic mutations in *JAM2*.[15] They interestingly show failure of JAM2 to translocate to the plasma membrane in JAM2 transfected mutants of hamster ovary cells, and they propose a cell-to-cell adhesion impairment as the mechanism causing failure of the NVU and consequent brain calcification phenotype. This work further supports our hypothesis and together with *PDGFB*, *PDGFRB*, and *MYORG* the tight junction genes solidify dysregulation of BBB integrity as a key PFBC pathomechanism (Figure S7).

Importantly, we show that the human *JAM2*-related neurological phenotype seen is replicated in the *jam2* KO mouse. It is noteworthy that the older human individuals carrying *JAM2* variants, who had longer disease duration, presented impaired gait of variable severity including ataxic and/or parkinsonian gait illustrated in the mouse model. The main walking and behavioral findings in the affected mice were progressive gait abnormalities, similar to ataxic mouse, clearly seen on standard mouse phenotype assessment. Furthermore, neuropathology of *jam2* KO mouse model (prominent vacuolation in the cerebral cortex, thalamus, and cerebellar cortex and particularly widespread vacuolation in the midbrain) affected similar brain areas to those observed on brain imaging of the human phenotype. The pattern of calcification seen on the brain scans of our subjects was largely the same as that seen in PFBC-affected subjects where the brain is structurally normal and the calcification is almost exclusively in the gray matter affecting basal ganglia, thalamus, deep cortex, dental, and cerebellar folia.[16] It was indistinguishable from *SLC20A2*, *PDGFB*, *PDGFRB*, and *XPR1* case subjects and there was no calcification in the pons as seen in *MYORG* subjects.[1,17] In contrast, the calcification pattern observed in *JAM2* subjects differs to that of other tight junction genes, as *OCLN* subjects have band-like calcification with simplified gyration[6] and *JAM3* subjects present multifocal intraparenchymal hemorrhage, massive cysts, and subependymal calcification.[5]

In summary, we show that *JAM2* is recurrently mutated in families with recessive PFBC presenting with a combination of movement disorder and/or cognitive and psychiatric manifestations. The human phenotype was replicated in a *jam2* KO mouse model. The presence of mutations in several genes involved in the central NVU presenting clinically with brain calcification suggests that the NVU is likely to represent a potential therapeutic target in this group of disorders.

Accession Numbers

The variants reported in this paper have been submitted to the Leiden Open Variation Database and the accession numbers are 643210, 643209, 643163, 643162.

Supplemental Data

Supplemental Data can be found online at https://doi.org/10.1016/j.ajhg.2020.02.007.

Acknowledgments

We are grateful to the patients for their essential help. We are grateful for the funding support from The Medical Research Council (MRC) (MR/S01165X/1, MR/S005021/1), The Wellcome Trust (Synaptopathies Strategic Award, WT093205MA, WT104033AIA), The Rosetree Trust, Ataxia UK, The MSA Trust, Brain Research UK, Sparks GOSH Charity, Muscular Dystrophy UK (MDUK), and Muscular Dystrophy Association (MDA USA). We also thank James Polke and Conceição Bettencourt for assistance with the text and interpretation. S.B. and H.H. are partly funded by the National Institute of Health Research (NIHR) UCLH/UCL Biomedical Research Centre and Dementia Biomedical Research Unit. J.R.M.deO. receives funding from CNP, CAPES, and DECIT-MS. We thank Joanne Lau for technical support. This research was

cortex (c), cerebellar cortex (c1), and midbrain (c2) (H&E). There is mild patchy astrogliosis in the midbrain (c3) and mild microglial activation (c4). No significant disruption of the cytoarchitecture is seen on APP immunostaining (c5), but NeuN highlights some degree of neuronal loss (c6). In age-matched old *jam2* KO mice, there is increasingly prominent vacuolation in the cerebral cortex (d) and in the cerebellar white matter (d1), midbrain (d2), and thalamus (not shown). Similar to young *jam2* KO mice, there is widespread astrogliosis in the midbrain (d3), but microglial activation remains mildly increased (d4). Frequent vacuolation is highlighted in the neuropil with APP immunostaining (d5). Similar to age-matched littermate controls, there is reduction of the neuronal density in the midbrain of *jam2* KO mouse (d6). Scale bar: 100 μm in a–d, a2–a6, b2–b6, c2–c6, d2–d6; 200 μm in a1–d1.

also conducted as part of the Queen Square Genomics group at UCL, supported by the National Institute for Health Research (NIHR) award to UCLH NHS Trust/UCL Biomedical Research Centre (BRC).

Declaration of Interests

The authors declare that A. Begtrup and E.T. are employees of GeneDx, Inc., USA, and S.K. and C.B. are employees of CENTOGENE AG, Rostock, Germany. The other authors declare no competing interests.

Received: October 9, 2019
Accepted: February 10, 2020
Published: March 5, 2020

Web Resources

CADD, https://cadd.gs.washington.edu/
Ensembl Genome Browser, http://www.ensembl.org/index.html
ExAC Browser, http://exac.broadinstitute.org/
GeneMatcher, https://genematcher.org/
GERP, http://mendel.stanford.edu/sidowlab/downloads/gerp/index.html
gnomAD Browser, https://gnomad.broadinstitute.org/
HomozygosityMapper software, http://www.homozygositymapper.org/
Leiden Open Variation Database, https://www.lovd.nl/
Mutation Assessor, http://mutationassessor.org/
MutationTaster, http://www.mutationtaster.org/
OMIM, https://www.omim.org/
PolyPhen-2, http://genetics.bwh.harvard.edu/pph2/
PROVEAN, http://provean.jcvi.org
QuPath, https://qupath.github.io/
SIFT, https://sift.bii.a-star.edu.sg/

References

1. Batla, A., Tai, X.Y., Schottlaender, L., Erro, R., Balint, B., and Bhatia, K.P. (2017). Deconstructing Fahr's disease/syndrome of brain calcification in the era of new genes. Parkinsonism Relat. Disord *37*, 1–10.
2. Taglia, I., Bonifati, V., Mignarri, A., Dotti, M.T., and Federico, A. (2015). Primary familial brain calcification: update on molecular genetics. Neurol. Sci. *36*, 787–794.
3. Legati, A., Giovannini, D., Nicolas, G., López-Sánchez, U., Quintáns, B., Oliveira, J.R.M., Sears, R.L., Ramos, E.M., Spiteri, E., Sobrido, M.-J., et al. (2015). Mutations in XPR1 cause primary familial brain calcification associated with altered phosphate export. Nat. Genet. *47*, 579–581.
4. Yao, X.-P., Cheng, X., Wang, C., Zhao, M., Guo, X.-X., Su, H.-Z., Lai, L.-L., Zou, X.-H., Chen, X.-J., Zhao, Y., et al. (2018). Biallelic Mutations in MYORG Cause Autosomal Recessive Primary Familial Brain Calcification. Neuron *98*, 1116–1123.e5.
5. Mochida, G.H., Ganesh, V.S., Felie, J.M., Gleason, D., Hill, R.S., Clapham, K.R., Rakiec, D., Tan, W.-H., Akawi, N., Al-Saffar, M., et al. (2010). A homozygous mutation in the tight-junction protein JAM3 causes hemorrhagic destruction of the brain, subependymal calcification, and congenital cataracts. Am. J. Hum. Genet. *87*, 882–889.
6. O'Driscoll, M.C., Daly, S.B., Urquhart, J.E., Black, G.C.M., Pilz, D.T., Brockmann, K., McEntagart, M., Abdel-Salam, G., Zaki, M., Wolf, N.I., et al. (2010). Recessive mutations in the gene encoding the tight junction protein occludin cause band-like calcification with simplified gyration and polymicrogyria. Am. J. Hum. Genet. *87*, 354–364.
7. Jensen, N., Schrøder, H.D., Hejbøl, E.K., Thomsen, J.S., Brüel, A., Larsen, F.T., Vinding, M.C., Orlowski, D., Füchtbauer, E.-M., Oliveira, J.R.M., and Pedersen, L. (2018). Mice Knocked Out for the Primary Brain Calcification-Associated Gene Slc20a2 Show Unimpaired Prenatal Survival but Retarded Growth and Nodules in the Brain that Grow and Calcify Over Time. Am. J. Pathol. *188*, 1865–1881.
8. Kimura, T., Miura, T., Aoki, K., Saito, S., Hondo, H., Konno, T., Uchiyama, A., Ikeuchi, T., Takahashi, H., and Kakita, A. (2016). Familial idiopathic basal ganglia calcification: Histopathologic features of an autopsied patient with an SLC20A2 mutation. Neuropathology *36*, 365–371.
9. Kobayashi, S., Yamadori, I., Miki, H., and Ohmori, M. (1987). Idiopathic nonarteriosclerotic cerebral calcification (Fahr's disease): an electron microscopic study. Acta Neuropathol. *73*, 62–66.
10. Miklossy, J., Mackenzie, I.R., Dorovini-Zis, K., Calne, D.B., Wszolek, Z.K., Klegeris, A., and McGeer, P.L. (2005). Severe vascular disturbance in a case of familial brain calcinosis. Acta Neuropathol. *109*, 643–653.
11. Keller, A., Westenberger, A., Sobrido, M.J., García-Murias, M., Domingo, A., Sears, R.L., Lemos, R.R., Ordoñez-Ugalde, A., Nicolas, G., da Cunha, J.E.G., et al. (2013). Mutations in the gene encoding PDGF-B cause brain calcifications in humans and mice. Nat. Genet. *45*, 1077–1082.
12. Aurrand-Lions, M., Johnson-Leger, C., Wong, C., Du Pasquier, L., and Imhof, B.A. (2001). Heterogeneity of endothelial junctions is reflected by differential expression and specific subcellular localization of the three JAM family members. Blood *98*, 3699–3707.
13. Tietz, S., Périnat, T., Greene, G., Enzmann, G., Deutsch, U., Adams, R., Imhof, B., Aurrand-Lions, M., and Engelhardt, B. (2018). Lack of junctional adhesion molecule (JAM)-B ameliorates experimental autoimmune encephalomyelitis. Brain Behav. Immun. *73*, 3–20.
14. Redmond, S.A., Mei, F., Eshed-Eisenbach, Y., Osso, L.A., Leshkowitz, D., Shen, Y.-A.A., Kay, J.N., Aurrand-Lions, M., Lyons, D.A., Peles, E., and Chan, J.R. (2016). Somatodendritic Expression of JAM2 Inhibits Oligodendrocyte Myelination. Neuron *91*, 824–836.
15. Cen, Z., Chen, Y., Chen, S., Wang, H., Yang, D., Zhang, H., Wu, H., Wang, L., Tang, S., Ye, J., et al. (2020). Biallelic loss-of-function mutations in JAM2 cause primary familial brain calcification. Brain *143*, 491–502.
16. Livingston, J.H., Stivaros, S., Warren, D., and Crow, Y.J. (2014). Intracranial calcification in childhood: a review of aetiologies and recognizable phenotypes. Dev. Med. Child Neurol. *56*, 612–626.
17. Grangeon, L., Wallon, D., Charbonnier, C., Quenez, O., Richard, A.-C., Rousseau, S., Budowski, C., Lebouvier, T., Corbille, A.-G., Vidailhet, M., et al.; French PFBC study group (2019). Biallelic MYORG mutation carriers exhibit primary brain calcification with a distinct phenotype. Brain *142*, 1573–1586.

The American Journal of Human Genetics, Volume *106*

Supplemental Data

Bi-allelic *JAM2* Variants Lead to Early-Onset Recessive Primary Familial Brain Calcification

Lucia V. Schottlaender, Rosella Abeti, Zane Jaunmuktane, Carol Macmillan, Viorica Chelban, Benjamin O'Callaghan, John McKinley, Reza Maroofian, Stephanie Efthymiou, Alkyoni Athanasiou-Fragkouli, Raeburn Forbes, Marc P.M. Soutar, John H. Livingston, Bernardett Kalmar, Orlando Swayne, Gary Hotton, SYNAPS Study Group, Alan Pittman, João Ricardo Mendes de Oliveira, Maria de Grandis, Angela Richard-Loendt, Francesca Launchbury, Juri Althonayan, Gavin McDonnell, Aisling Carr, Suliman Khan, Christian Beetz, Atil Bisgin, Sevcan Tug Bozdogan, Amber Begtrup, Erin Torti, Linda Greensmith, Paola Giunti, Patrick J. Morrison, Sebastian Brandner, Michel Aurrand-Lions, and Henry Houlden

Supplemental data

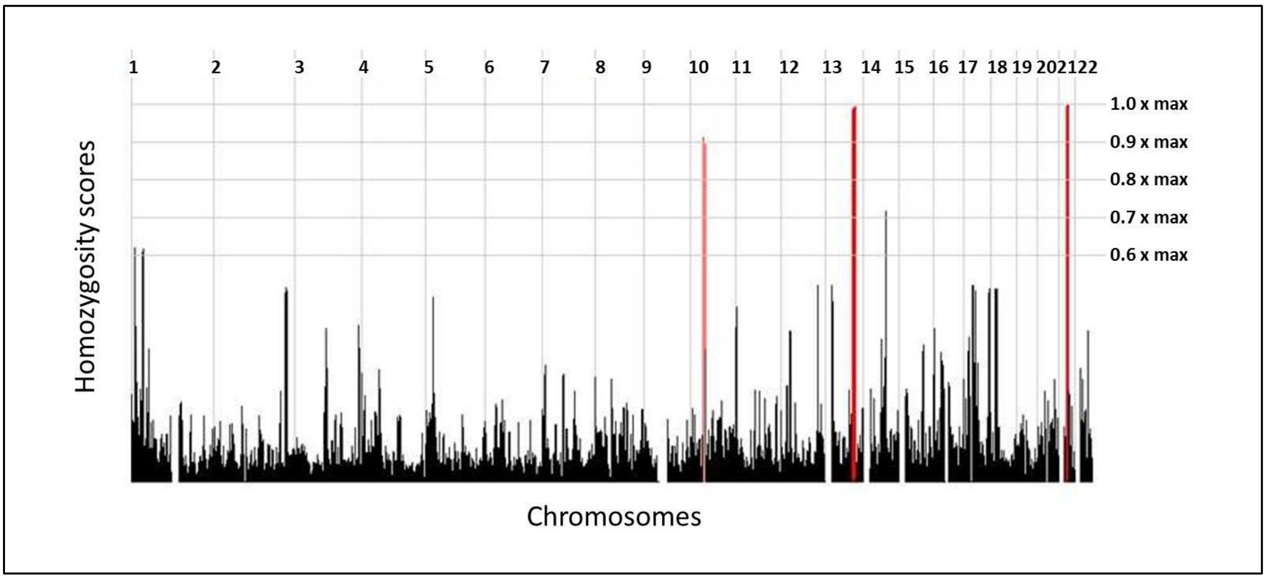

Figure S1: Genome wide illustration of homozygous regions. Three significant regions of homozygosity were detected: Chr 10: 37414883-43132376; Chr 13: 88327643-93518692; and Chr 21: 22370881-28338710 and they are highlighted in red.

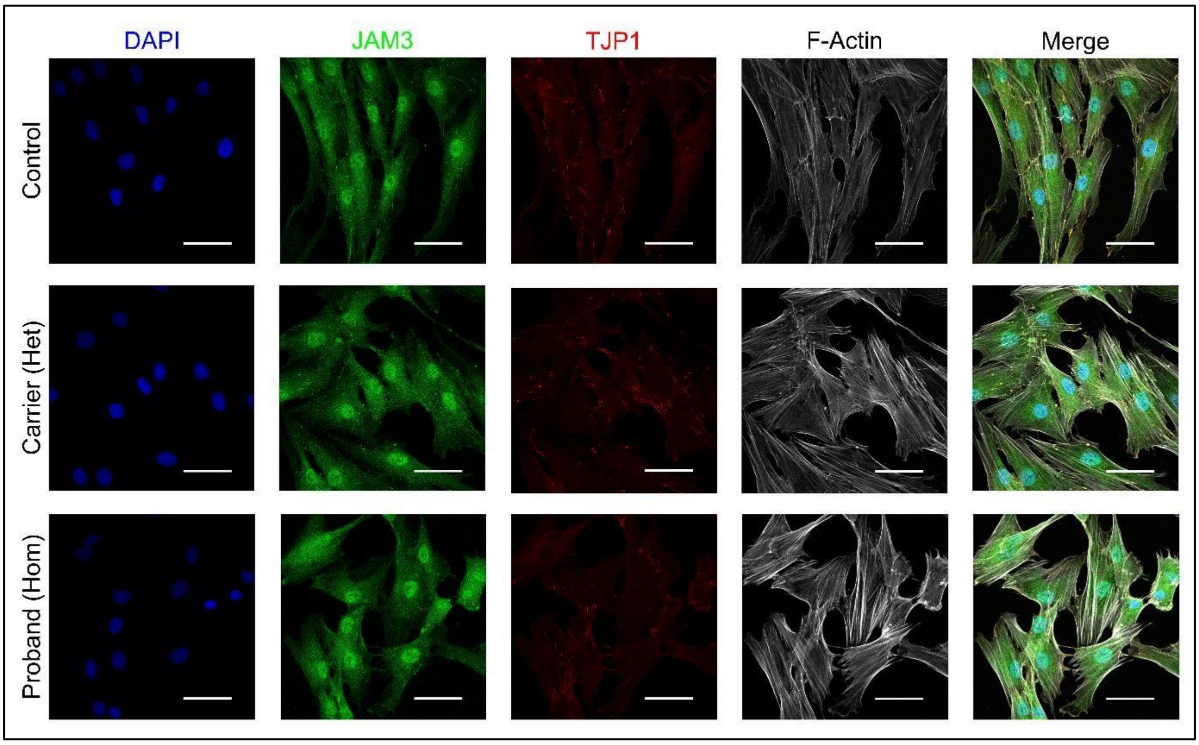

Figure S2: Localisation of JAM3 and TJP1 tight junction proteins in primary dermal fibroblasts from the proband of Family 1 (Hom) and his unaffected mother (Het). Scale bars = 50 μm.

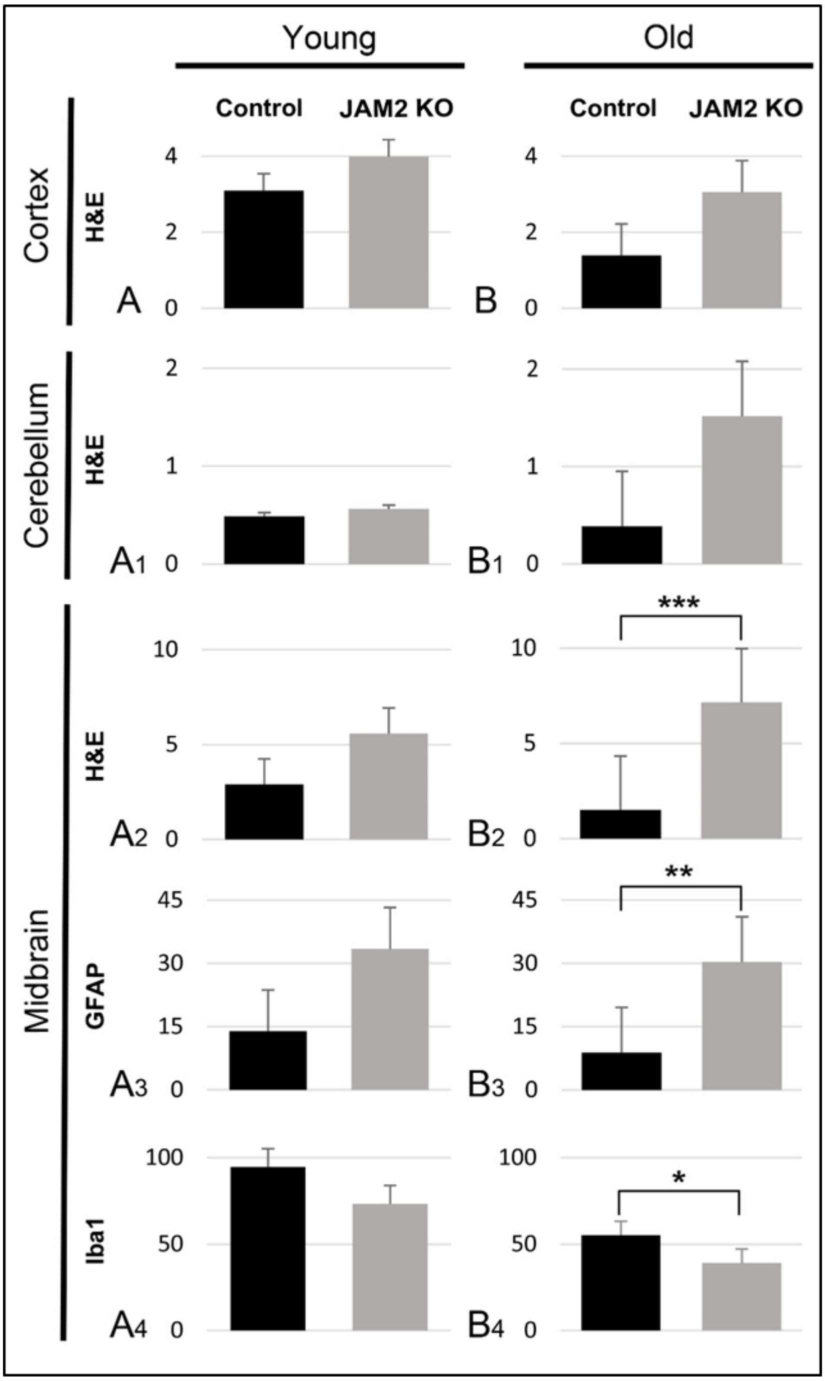

Figure S3: Quantitative data of neuropil vacuolation, astrogliosis and microglial activation in young and old *jam*2 KO and wild-type (Control) mice. The automated quantification of the neuropil vacuolation on H&E stained sections (A, A1, A2, B, B1, B2), GFAP immunoreactive gliosis (A3, B3) and Iba1 positive microglial activation (A4, B4) in young (A-A4) and aged (B-B4) wild-type (black columns) and *jam2* KO (grey columns) mice. Automated quantification of the percentage of vacuolation, gliosis and microglial activation in

selected areas (cortex, midbrain and cerebellum) was performed on digitalised slides, using open source software QuPath.

In young *jam2* KO mice (n=2) when compared with age-matched wild-type mice (n=2), there was a trend towards greater neuropil vacuolation in the cortex (A), cerebellum (A1) and midbrain (A2). Similarly, GFAP positive astrogliosis (A3), but not Iba1 positive microglial activation (A4), was more prominent in young *jam2* KO mice, than wild-type mice. In the aged *jam2* KO mice (n=4), there was a trend towards greater neuropil vacuolation in the cortex (B) and cerebellum (B1), when compared with age-matched wild-type mice (n=4). There was a significant increase in the degree of neuropil vacuolation (*** $p<0.00007$) and astrogliosis (**$p<0.0138$) in the midbrain (B3) of old *jam2* KO mice when compared with age-matched wild-type mice, whereas Iba1 positive microglial activation (B4) in old *jam2* KO mice was less pronounced than in age-matched wild-type mice (*$p<0.035$).

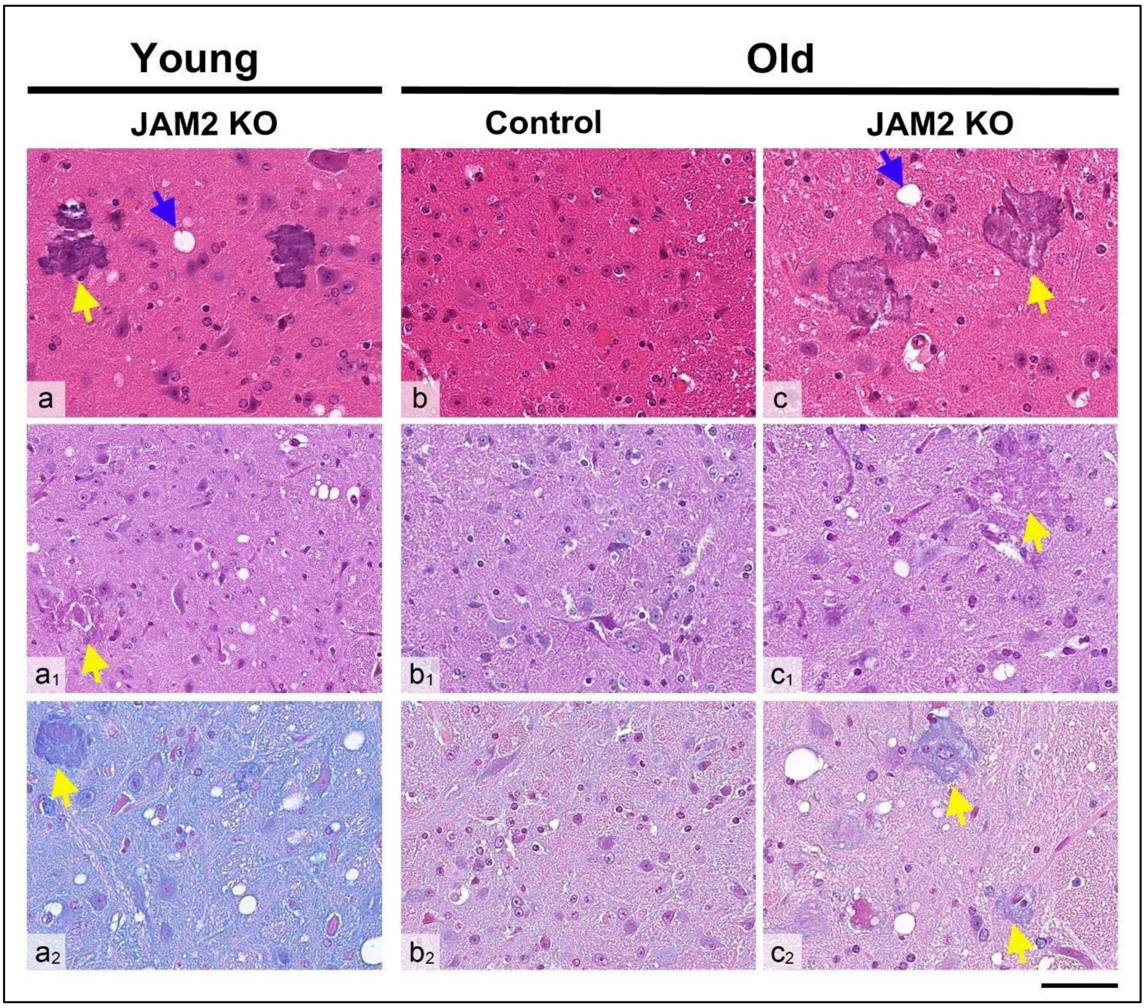

Figure S4: Spinal cord pathology in control mice and *jam2* KO mice of young (6 months) and old (18 months) age. In the grey matter of young *jam2* KO mice (a, a1 and a2) (a1) there is frequent mineralisation (yellow arrow) and occasional vacuolation in the neuropil (blue arrow). The mineralised deposits show no reactivity with PAS tinctorial stain (a1, yellow arrow) and very weak labelling with Alcian blue (a2, yellow arrow). In the spinal cord grey matter of old control wild-type mice (b, b1 and b2) there is no mineralisation and no apparent neuropil vacuolation. In the spinal cord of an old *jam2* KO mouse (c, c1 and c2), however, there is frequent mineralisation (c, yellow arrow) and vacuolation in the neuropil (blue arrow) involving both anterior and posterior horns. The mineralised deposits also in old *jam2* KO mice are not positive for PAS (c1) and weakly label for Alcian blue (c2, one yellow arrow

accentuates a mineralised neurone and the other shows perivascular mineralisation). Scale bar: 100µm a-c, a1-c1, a2-c2.

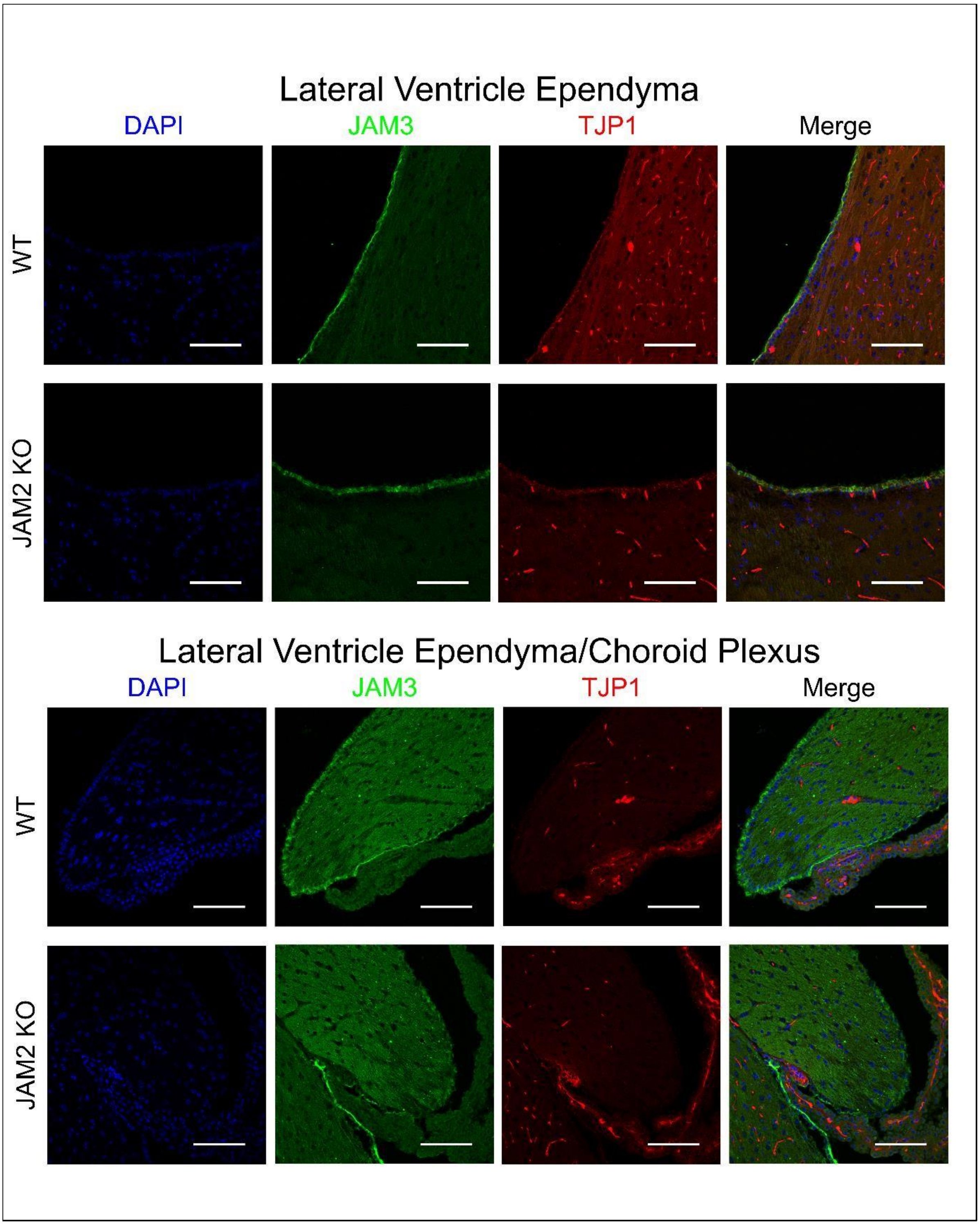

Figure S5: Correct localisation of JAM3 and TJP1 tight-junction proteins in the lateral ventricle ependyma of *jam2* KO and wild-type (WT) mice. Scale bars = 100 μm.

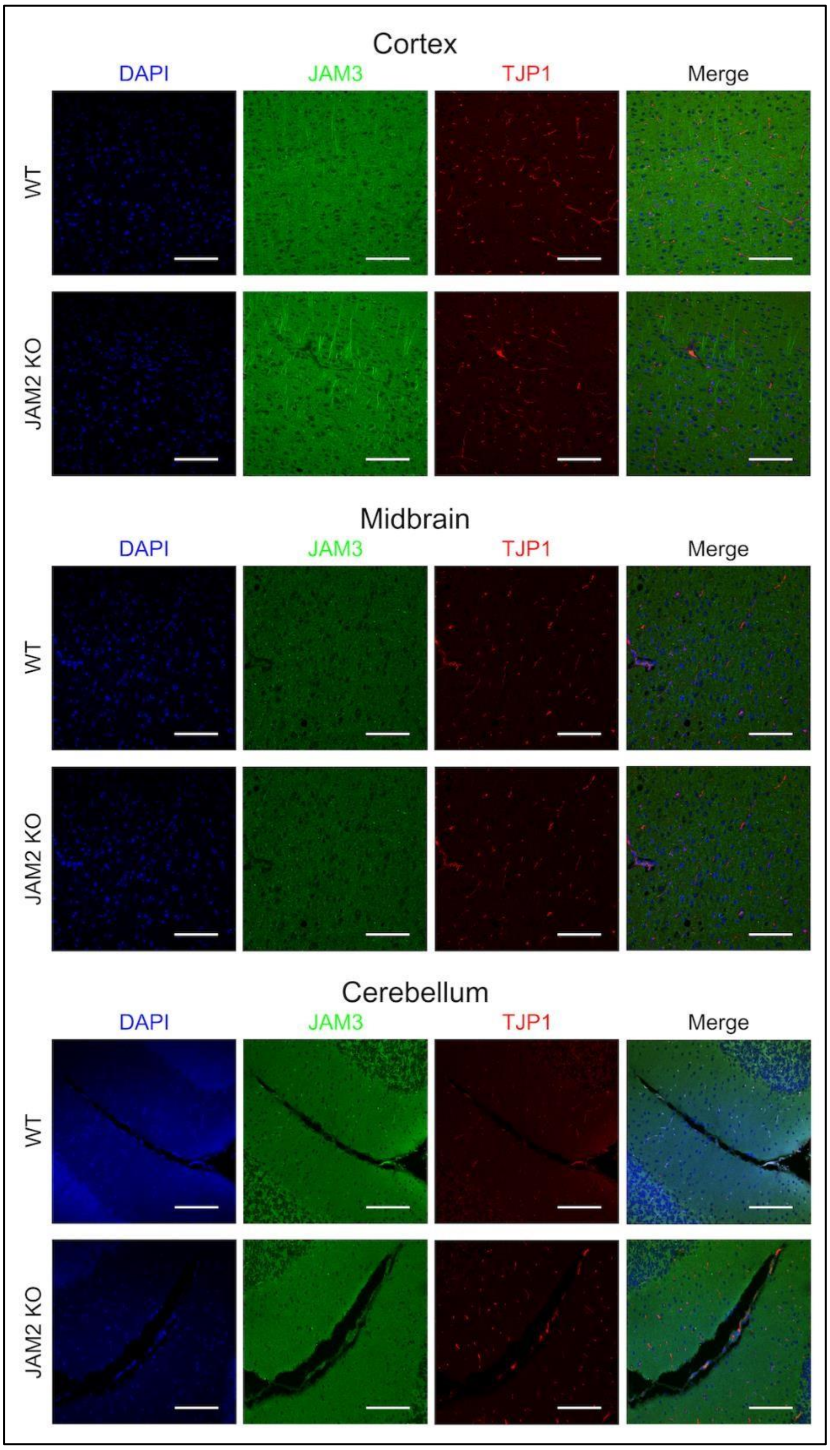

Figure S6: Unaltered staining pattern for JAM3 and TJP1 tight-junction proteins in different brain regions of *jam2* KO and wild-type (WT) mice. Scale bars = 100 µm.

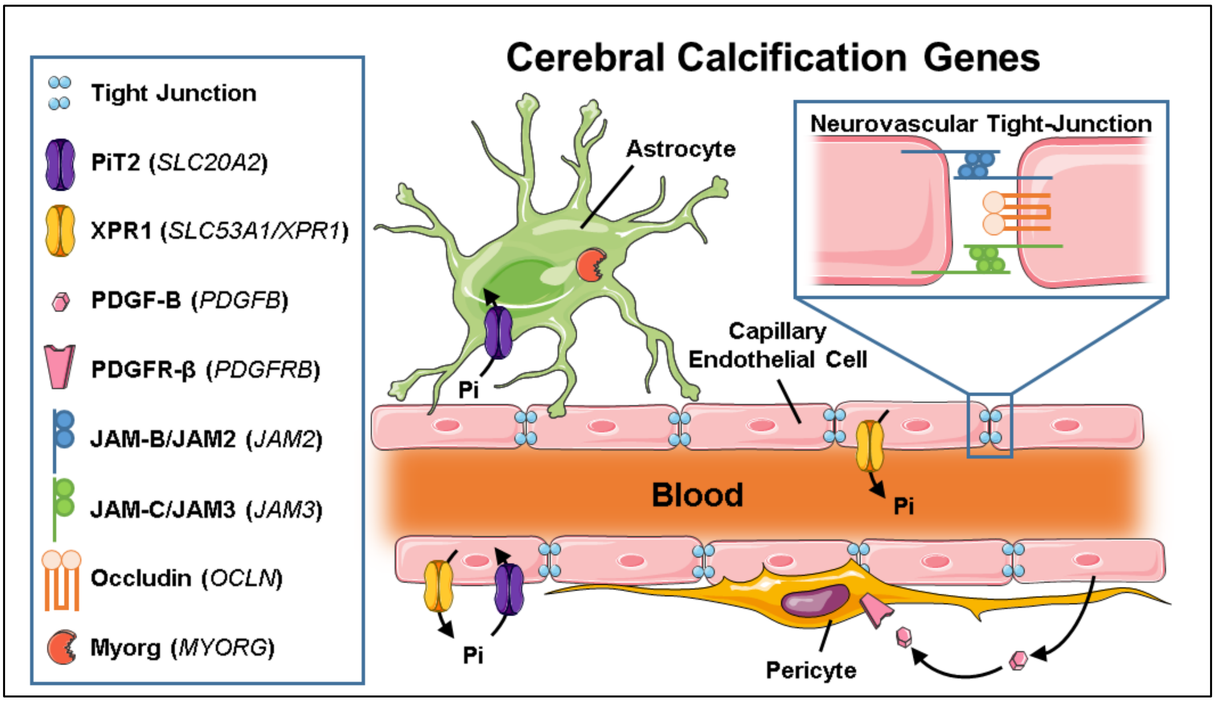

Figure S7: Schematic highlighting the contributions of proteins associated with brain calcification disorders in cell types of the central neurovascular unit (NVU). Autosomal dominant mutations in genes encoding the inorganic phosphate transporter 2; (PiT2 encoded by *SLC20A2*) and inorganic phosphate exporter; (XPR1 encoded by *SLC53A1/XPR1*) are causative of PFBC, highlighting dysregulation of phosphate homeostasis as an important cause of calcium deposition. Platelet-derived growth factor subunit B (PDGF-B encoded by *PDGFB*) signalling through the platelet-derived growth factor receptor-β (PDGFR-β encoded by *PDGFRB*) is important for pericyte survival and blood brain barrier (BBB) maintenance. Autosomal dominant mutations in *PDGFB* and *PDGFRB* cause PFBC suggesting loss of BBB integrity as an additional pathomechanism. In line with this, recessive mutations in *JAM3* and *OCLN* encoding tight junction proteins have been associated with complex neurological disorders that include brain calcification. The *JAM2* mutations identified in this study further support dysregulation of BBB integrity as a PFBC pathomechanism. This figure was prepared using Servier Medical Art, licensed under a Creative Common Attribution 3.0 Generic License (http://smart.servier.com/).

Supplemental methods

This study was approved by UCLH Queen Square ethics committee and local institutional review board (UCLH: 04/N034) and informed consent was obtained from all subjects or their legal guardians when applicable.

Genome-wide genotyping and homozygosity mapping

Genomic DNA was extracted from peripheral blood samples. In family 2 genome-wide genotyping was performed according to the manufacturers' instructions on Illumina HumanCytoSNP-12v2-1_H array, which contains probes for over 200,000 markers distributed across the genome. Default parameters on genome studio software 2010.3 (Illumina) were used for clustering and normalization of raw data, and to obtain genotype calls. Regions of homozygosity were then analysed with homozygosity mapper software[1].

Exome sequencing (ES)

ES was performed in all families. In familied 1 and 2 exome sequencing was done with Illumina® Nextera focused exome capture on a HiSeq 2000. Paired-end sequence reads were aligned with Novoalign against the reference human genome (UCSC hg19). Duplicate read removal, format conversion, and indexing were performed with Picard. The Genome Analysis Toolkit (GATK) was used to recalibrate base quality scores, perform local realignments around possible indels, and to call and filter the variants. Annotated variant files were generated using ANNOVAR[2]. ES in family 3 was carried by GeneDx. Using genomic DNA from the proband and parents, the exonic regions and flanking splice junctions of the genome were captured using the Clinical Research Exome kit (Agilent Technologies, Santa Clara, CA). Massively parallel (NextGen) sequencing was done on an Illumina system with 100bp or greater paired-end reads. Reads were aligned to human genome build GRCh37/UCSC hg19, and analyzed for sequence

variants using a custom-developed analysis tool. Additional sequencing technology and variant interpretation protocol has been previously described[3]. The general assertion criteria for variant classification are publicly available on the GeneDx ClinVar submission page (http://www.ncbi.nlm.nih.gov/clinvar/submitters/26957/). ES in the proband of family 4 was performed using the capture system from Twist Human Core Exome Plus kit (Twist Bioscience®) and sequenced on a Illumina HiSeqX that produced 150-bp paired-end reads. Sequencing data were aligned using BWA software and alignments were converted to binary bam file format with GATK. For variant calling we utilized the GATK HaplotypeCaller. Additional sequencing and bioinformatics information on this case was previously described[4].

Cell culture

Fibroblasts from one proband and his mother from Family 1 and unrelated controls were cultured from a skin biopsy in Dulbecco's modified Eagle medium containing 4.5 g/L glucose and supplemented with 10 % heat-inactivated fetal calf serum and maintained in a humidified chamber at 37 °C with 5 % CO2.

RT-PCR

RNA was extracted from human fibroblasts and purified using the Quick-RNA Miniprep Kit (Zymo Research). A complementary DNA (cDNA) library was immediately created from 500 ng of the RNA obtained, by reverse transcription reaction with Superscript II reverse transcriptase (Thermofisher). Reverse transcription PCR (RT-PCR) was then performed using equal volumes of cDNA using FastStart Taq DNA polymerase master mix and primer pairs specific for GAPDH (loading control) and *JAM2* (Supplementary Table 1). Equal volumes of the PCR mixes were then separated by DNA gel electrophoresis through 1.2% w/v agarose TAE. Dye-terminator sequencing reactions were performed using the BigDye® Terminator

v3.1 Cycle Sequencing Kit (Applied Biosystems) and analysed on a 3730xl DNA Analyser (Applied Biosystems).

The primers used for RT-PCR were:

**Target**	**Forward Primer (5’-3’)**	**Reverse Primer (5’-3’)**	**Product (bp)**
GAPDH	TGGTGCTGAGTATGTCGTGGAGT	AGTCTTCTGAGTGGCAGTGATGG	292
JAM2	GTGACAAGAAGTGATGCGGG	GCATAGCATACACCAAGGCC	488

SDS-PAGE and Western blotting

Cell lysates were prepared by washing cells with cold PBS, followed by lysis buffer addition (50 mM Tris, 0.1 mM EGTA, 1 mM EDTA, 0.27 M sucrose, 1 % Triton X-100, Protease Inhibitor Cocktail (Roche) Phosphatase Inhibitor Cocktail (Sigma). Cell lysates were then collected and clarified by centrifugation at 12,500 rpm for 10 minutes at 4°C. Proteins were separated on Novex precasted SDS-PAGE Bis-Tris 4-12% gels (Invitrogen), using MES running buffer (Invitrogen). Proteins were then transferred to PVDF membranes (Millipore), blocked with 5 % milk and blotted with JAM2 (ThermoFisher PA5-21576) and GAPDH as loading control.

Animal studies

The mouse strain used in this study is Jam2$^{tm1.2Rha}$ for both *jam2* (encoding for Jam-B) knockout (KO) and wild-type (WT) mice. Mice were housed in the animal facility and were maintained on a 12-hour light dark cycle at a constant temperature and humidity. All animal experiments were carried out according to the UK Animal Act 1986 and approved by the UCL Animal Care Committee.

Generation of jam-2 deficient mice

The generation of this colony was previously described in the literature[5]. Briefly, a *jam2* (ortholog of human *JAM2*) gene fragment containing exon 5 was flanked by loxP recognition sites for Cre recombinase. Within the loxP-flanked region, a murine *jam2* cDNA fragment (codons 165-298, PEY…SFII*) followed by the bovine growth hormone polyadenylation signal (pA) was fused to a BspE1 site in exon 5. The construct also contained a neomycin resistance cassette surrounded by frt sites enabling Flp recombinase-mediated removal, as well as long (9 kb, 5′) and short (1.65 kb, 3′) arms for homologous recombination. Electroporated and G418-selected embryonic stem cell clones were analysed by Southern blot hybridization and polymerase chain reaction (PCR). For PCR screening, a primer pair, derived from exon 4 and the intronic *jam-2* sequence flanking the 5' loxP site, amplified a 345 bp band from WT chromosomes and a 450bp band from transgenic chromosomes (primers were 5′-AGACCGTGCTGAGATGATAGA-3′ and 5′-CCGAAGGAAGTGTCTAGTAAT-3′). Three independent lines were generated and maintained in a mixed 129Sv × C57BL/6 background. *jam-2* deficient mutants were generated by crossbreeding with the PGK-Cre line followed by interbreeding *jam2* KO/+ heterozygotes. Mice used in the present study were backcrossed for more than 12 generations on C57Bl/6 background.

Behavioural studies

Behavioural studies were performed in the *jam2* KO mice and compared to controls as described[6]. All mice were trained before the relevant tests. The balance beam walking test assesses mouse's ability to maintain balance while traversing a narrow beam to reach a safe platform by counting and comparing the number of missed steps between *jam2* KO and control mice. The footprint analysis assesses motor coordination and synchrony by examining gait during normal walking. The fore and hind paws were painted with dyes of different colours and the mice were encouraged to walk in a straight line over absorbent paper. Stance, stride

and sway measurements were compared among groups. A Mann-Whitney test (means ± SEM) was used to analyse the results of the behavioural tests.

Histological analysis

Mice were culled by injection of a pentobarbital overdose. The brains and the spinal cords were harvested and fixed in formalin. After formalin fixation the brains were cut sagittaly and the cervical, thoracic and lumbar spinal cord segments were cut transversely and processed for paraffin histology. The 5µm thin sections of the tissue were stained with haematoxylin and eosin (H&E) for routine examination. Representative sections from each brain and spinal cord were further assessed with Alcian Blue (Alcian Blue: Sigma, C174240; Gill III Haematoxylin: Thermo Shandon, 6765009, Schiffs: VWR, 351204L) and PAS (Ventana PAS Kit, Ventana 860-014) tinctorial stains and with immunohistochemical stains for Iba1 (Wako, 019-19741, 1:250), GFAP (Agilent, Z0334, 1:1000), NeuN (Chemicon, MAB377, 1:2000) and APP (Millipore, MAB348, 1:800). The Alcian Blue was hand-stained. The PAS tinctorial stain and immunostainings were performed on a ROCHE Ventana Discovery automated staining platform following the manufacturer's guidelines. Biotinylated secondary antibodies and a horseradish peroxidase-conjugated streptavidin complex and diaminobenzidine as a chromogen were used for the immunostainings. All staining was carried out with appropriate controls.

Automated quantification of histological results

For image analysis and automated pathology quantification, histological slides were first digitised on a LEICA SCN400F scanner (LEICA Milton Keynes, UK) at x40 magnification and 65% image compression setting during export. Then, from whole digitalised slides, the full thickness of cortex, entire midbrain and entire cerebellar white matter with cortical granule cell layer was manually selected for quantification studies. The percentage of neuropil vacuolation

on H&E stained sections, astrogliosis on GFAP immunostained sections and microglial activation on Iba1 immunostained slides in the manually selected cortical, midbrain and cerebellar regions was quantified, using an algorithm developed on open source digitalised image analysis software QuPath (v.0.2.0-m8)[7]. The difference of analysed parameters between wild-type and *jam2* KO mice in both young and old cohorts was analysed in Excel using paired t-test (with unequal variances), with $p < 0.05$ considered statistically significant.

Immunofluorescence analysis

Fibroblasts were fixed with 4% v/v formaldehyde in Dulbecco's Phosphate-Buffered Saline (DPBS) for 15 min at room temperature (RT). Slices of mouse brain were prepared as descried above for histological analysis. Cells/brain slices were incubated in blocking and permeabilisation solution (BPS) consisting of: 10% v/v donkey serum, 0.1% w/v triton-x100 in DPBS for 1 hr. Cells/slices were then incubated with primary antibodies diluted in BPS targeting JAM3/JAM-C (goat anti-JAM-C, RnD Systems AF1213, 10μg/ml), and TJP1 (Mouse anti-ZO-1, Thermofisher Scientific 339100, 5μg/ml) for 2 hrs. This was followed by incubation with donkey anti-goat Alex Fluor-488 (Thermofisher A11055, 1:1000) and donkey anti-mouse Alex Fluor-568 (Thermofisher a10037, 1:1000) fluorescently conjugated antibodies, and phalloidin-647 for 1 hr. Nuclei were counterstained with 0.1 μg/ml 4′,6-diamidino-2phenylindole (DAPI) and mounted with mounting medium (Dako) for confocal microscopy. Images were acquired with a Zeiss 710 VIS CLSM equipped with a META detection system using 20x and 63x (oil immersion) objectives, and max intensity projections prepared using ImageJ FIJI software.

References

1. Seelow, D., Schuelke, M., Hildebrandt, F., and Nürnberg, P. (2009). HomozygosityMapper--an interactive approach to homozygosity mapping. Nucleic Acids Res. *37*, W593-599.

2. Wang, K., Li, M., and Hakonarson, H. (2010). ANNOVAR: functional annotation of genetic variants from high-throughput sequencing data. Nucleic Acids Res. *38*, e164.

3. Retterer, K., Juusola, J., Cho, M.T., Vitazka, P., Millan, F., Gibellini, F., Vertino-Bell, A., Smaoui, N., Neidich, J., Monaghan, K.G., et al. (2016). Clinical application of whole-exome sequencing across clinical indications. Genet. Med. *18*, 696–704.

4. Bauer, P., Kandaswamy, K.K., Weiss, M.E.R., Paknia, O., Werber, M., Bertoli-Avella, A.M., Yüksel, Z., Bochinska, M., Oprea, G.E., Kishore, S., et al. (2019). Development of an evidence-based algorithm that optimizes sensitivity and specificity in ES-based diagnostics of a clinically heterogeneous patient population. Genet. Med. *21*, 53–61.

5. Arcangeli, M.-L., Frontera, V., Bardin, F., Obrados, E., Adams, S., Chabannon, C., Schiff, C., Mancini, S.J.C., Adams, R.H., and Aurrand-Lions, M. (2011). JAM-B regulates maintenance of hematopoietic stem cells in the bone marrow. Blood *118*, 4609–4619.

6. Brooks, S.P., and Dunnett, S.B. (2009). Tests to assess motor phenotype in mice: a user's guide. Nat. Rev. Neurosci. *10*, 519–529.

7. Bankhead, P., Loughrey, M.B., Fernández, J.A., Dombrowski, Y., McArt, D.G., Dunne, P.D., McQuaid, S., Gray, R.T., Murray, L.J., Coleman, H.G., et al. (2017). QuPath: Open source software for digital pathology image analysis. Sci Rep *7*, 16878.
